# Unraveling temporal and spatial biomarkers of epithelial-mesenchymal transition in colorectal cancer: insights into the crucial role of immunosuppressive cells

**DOI:** 10.1186/s12967-023-04600-x

**Published:** 2023-11-08

**Authors:** Muhong Wang, Chunyu Deng, Cheng Yang, Mingze Yan, Haibo Lu, Yan Zhang, Honghao Liu, Zhekuan Tong, Jiaao Ma, Jiaming Wang, Yan Zhang, Jiahao Wang, Yuhong Xuan, Haiyue Cheng, Kai Zhao, Jiaqi Zhang, Cuicui Chai, Mingzhe Li, Zhiwei Yu

**Affiliations:** 1 Department of Colorectal Surgery, Harbin Medical University Cancer Hospital, Harbin, 150086 China; 2 School of Life Science and Technology, Harbin Institute of Technology, Harbin, 150080 China; 3 Digestive Disease Center, The Seventh Affiliated Hospital Sun Yat-Sen University, Shenzhen, 518107 China

**Keywords:** Colorectal cancer, Single-cell RNA-seq, Spatial transcriptomics, Tumor immune microenvironment, Epithelial-mesenchymal transition (EMT)

## Abstract

The occurrence and progression of tumors can be established through a complex interplay among tumor cells undergoing epithelial-mesenchymal transition (EMT), invasive factors and immune cells. In this study, we employed single-cell RNA sequencing (scRNA-seq) and spatially resolved transcriptomics (ST) to evaluate the pseudotime trajectory and spatial interactive relationship between EMT-invasive malignant tumors and immune cells in primary colorectal cancer (CRC) tissues at different stages (stage I/II and stage III with tumor deposit). Our research characterized the spatiotemporal relationship among different invasive tumor programs by constructing pseudotime endpoint-EMT-invasion tumor programs (EMTPs) located at the edge of ST, utilizing evolution trajectory analysis integrated with EMT-invasion genes. Strikingly, the invasive and expansive process of tumors undergoes remarkable spatial reprogramming of regulatory and immunosuppressive cells, such as myeloid-derived suppressor cells (MDSCs), tumor-associated macrophages (TAMs), regulatory T cells (Treg), and exhausted T cells (Tex). These EMTP-adjacent cell are linked to EMT-related invasion genes, especially the C-X-C motif ligand 1 (CXCL1) and CXCL8 genes that are important for CRC prognosis. Interestingly, the EMTPs in stage I mainly produce an inflammatory margin invasive niche, while the EMTPs in stage III tissues likely produce a hypoxic pre-invasive niche. Our data demonstrate the crucial role of regulatory and immunosuppressive cells in tumor formation and progression of CRC. This study provides a framework to delineate the spatiotemporal invasive niche in CRC samples.

**Graphical Abstract:**

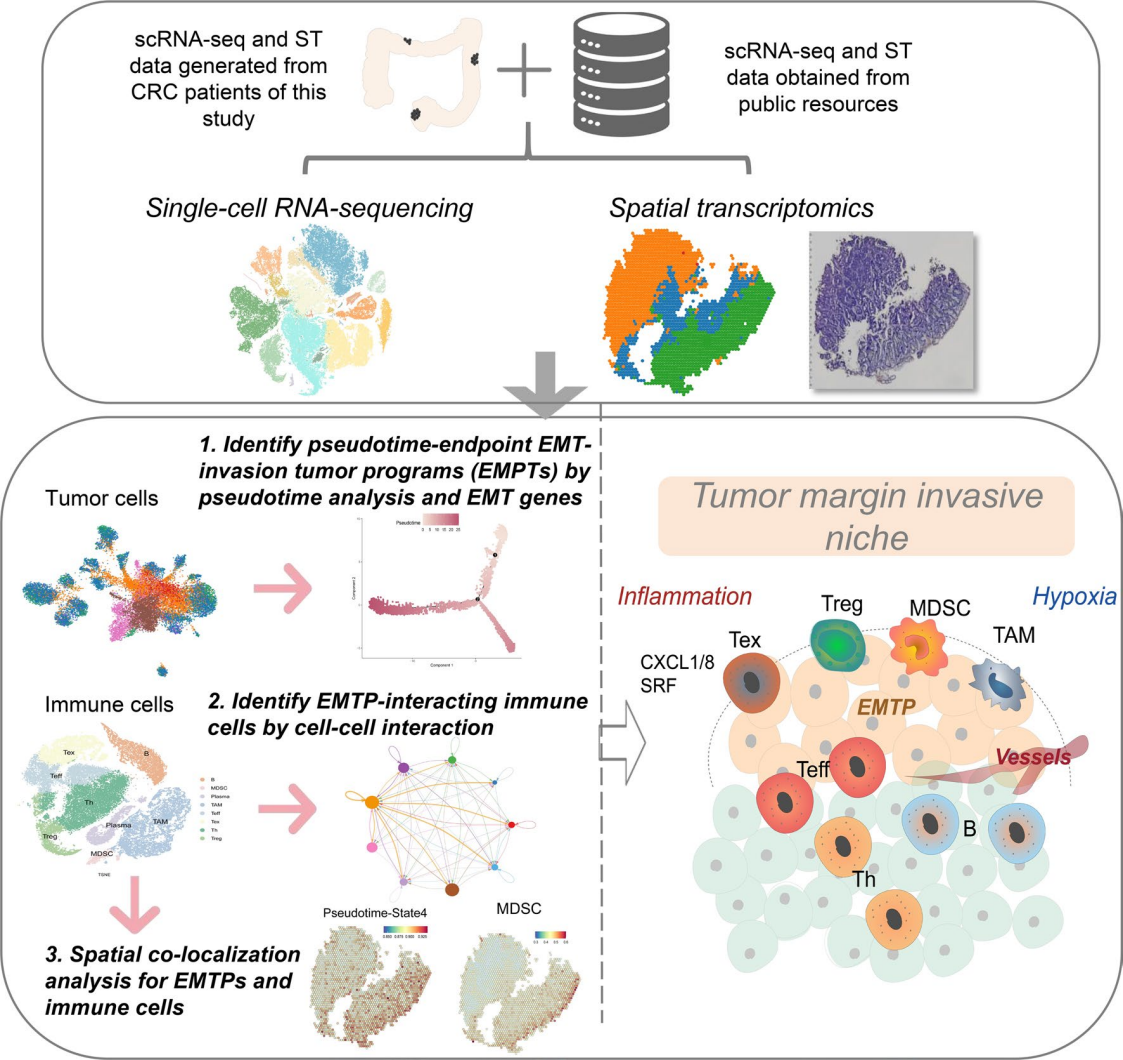

**Supplementary Information:**

The online version contains supplementary material available at 10.1186/s12967-023-04600-x.

## Introduction

Colorectal cancer (CRC) is one of the most significant health burdens worldwide [1], and 40% of the patients develop to the stage eventually leading to death [2]. Despite significant advances in understanding the molecular mechanisms underlying CRC development, including epigenetic regulation [3–6] and its pertinence to inflammation [7], our knowledge of CRC disease progression is still far from satisfactory.

The "seed and soil" hypothesis proposes that primary tumors can create a suitable microenvironment for tumor cell invasion before they arrive at distant sites, which has been instrumental in understanding tumor metastasis [8, 9]. We postulate that this hypothesis is equally applicable to the early-stage expansion process of tumors, particularly during their breach of the basement membrane and dissemination to lymph nodes. During the process of EMT invasion of tumor metastasis, the tumor immune microenvironment (TME) produces a highly immunosuppressive pre-metastasis niche [10] that induces a systemic loss of antigen-specific T lymphocytes [11], which allows tumor cells to invade adjacent normal tissues [12]. Similar to the concept of the pre-metastatic niche, a microenvironment conducive to tumor growth is established at the periphery before tumor development and expansion [13]. For the convenience of description, we term this microenvironment as the "margin invasive niche". However, the spatial coordination between immune cells and EMT-invasion tumor programs in primary CRC is largely unknown, and whether the EMT-invasion microenvironments are different in distinct tumor stages remains unclear [14]. Therefore, detection, identification and quantification of the temporal and spatial landscape during the EMT tumor invasion process in the early stages of primary CRC is increasingly necessary to advance our understanding of tumor growth.

ScRNA-seq allows us to determine the degree of tumor progression at a single-cell resolution through EMT-associated genes and provides a new perspective to interrogate the transcriptional heterogeneity of malignant cell subsets. In CRC research, scRNA-seq has been used to unravel the complexities of TME and tumor cell evolution [15–17]. On the other hand, spatial transcriptomics (ST) has also been employed to analyze RNA levels in a spatial context, shedding light on tissue and immune cell heterogeneity, and revealing subcellular RNA localization [18]. The understanding of tissue architecture aids in deciphering individual cell functions in multicellular organisms by pinpointing their precise physical locations within tissue Sections [19]. Moreover, integration of scRNA-seq with ST enables the systematic analysis and validation of the spatial locations and cell–cell interactions for multiple tumor programs in TME [20].

In this study, we utilized scRNA-seq and ST obtained from 42 samples to characterize the transcriptional landscape of CRC. Our findings reveal that the immune microenvironment of CRC exhibits spatiotemporal distribution in the process of EMT-invading tumors. Further analysis along pseudotime trajectory shows that tumor programs in both primary tumor stages shift towards more EMT-invasive and immunosuppressive stages where immunosuppressive cells are reprogrammed at invasion sites. Our study also highlights the role of recruited regulatory and suppressive immune cells, such as TAMs, MDSCs, Treg, and Tex cells, in promoting tumor progression by supporting the formation of margin invasive niches. Moreover, these cells are constructed for a variety of properties of niche formation at different stages.

## Material and methods

### Clinical sample collection

In this study, the scRNA-seq data were generated from two cases of CRC tissue samples, designated as RC1 and RC2, collected at the Seventh Affiliated Hospital of Sun Yat-sen University (Shenzhen, China), and the patients were diagnosed histologically. RC1 was collected from the periphery of the tumor exhibited invasion into the muscular layer without detectable lymph node metastasis, and was thus classified as stage I (T2N0M0). RC2 was also excised from tumor periphery showing invasion into extramural parietal fat and fibrous tissues of the rectum without distant lymph node metastasis, and was classified as stage III (T4N1cM0). Information regarding tissue dissociation and cDNA synthesis can be found in the Supplemental Methods.

### scRNA-seq data collection

Utilizing 10 × single-cell sequencing, we conducted analysis on the two CRC samples. To increase the comprehensiveness of our study, we incorporated publicly available scRNA-seq datasets (GSE132465, GSE132257, GSE144735 and E-MTAB-8107) into our analyses. These public datasets downloaded from GEO and ArrayExpress encompass 47 samples obtained from 40 patients at three different CRC stages, including stages I, II, and III, as shown in Additional file 1: Figure S1 and Additional file 12: Tables S1–S3. These additional cohorts were collected from early, treatment-naive patients using the 10XGenomics platform, and samples from late-stage patients with advanced distant metastasis (stage IV) not included.

### ST data collection

We employed the 10 × ST sequencing method to simultaneously capture the spatial data of RC1 and RC2. Furthermore, to corroborate our results, we obtained additional CRC-related ST data from a publicly accessible database of the CNGB Nucleotide Sequence Archive (CNSA: http://db.cngb.org, accession number CNP0002432).

### scRNA-seq data quality control and pre-processing

For the two CRC samples with both scRNA-seq and ST analyses conducted in this study, we processed the raw fastq data using Cell Ranger (v 3.1.0) with default arguments and the Homo_sapiens GRCh38 dataset as a reference file. To pre-process and control data quality, we mainly used the Seurat (v4.0.5) package to read and pre-process the data according to a previous study [21]. We first removed the cell data that expressed fewer than 300 genes or greater than > 20% mitochondrial genes and excluded the genes that were expressed in fewer than three cells. For the scRNA-seq data, we chose a relatively loose filtering standard to avoid excessive filtering of MDSCs, as they naturally express low RNA levels. This allowed us to retain more non-hematopoietic cells, which express many mitochondrial genes due to dissociation effects. We then normalized the unique molecular identifiers (UMIs) per cell and corrected the sequencing depth, mitochondrial gene percentage, rRNA percentage, G2M cycle, and other factors.

For the other publicly available data, we used Seurat to read all the count data from different sources and create a Seurat object with clinical information, mainly including CRC stages. After scaling the data as before, we selected 2,000 highly variable genes and merged all the single-cell data. Next, we produced principal component analysis (PCA) with npcs = 50. The Harmony package was used to eliminate the batch effect for the merged data of this study and the public data. Clustering analysis was carried out with a resolution of 0.2 on the merged scRNA-seq and ST data. We plotted the t-distributed stochastic neighbor embedding (t-SNE) for the data with 50 PCs. Clusters with less than 100 cells were excluded from the data analysis.

### Celltypes annotation

To obtain specific gene markers from different cell clusters, we separately calculated the differential genes for each cell type using the Wilcoxon rank sum test in the FindMarkers (*q* < 0.01). We first used the SingleR software based on a previous study [22] to annotate the cell types and determine approximate cell types using the celldex software package (Human Primary Cell Atlas Data; https://github.com/LTLA/celldex) (Additional file 12: Table S5). We then defined the names of cell types that accounted for the largest proportion of cell annotation result for each cluster. Furthermore, based on differentially expressed genes (Additional file 12: Table S6), we further verified the annotation results based on the top five differentially expressed genes determined by the Cellmarker (http://biocc.hrbmu.edu.cn/CellMarker/) [23]. Subsequently, to identify immune cell subsets in high resolution, we re-clustered the immune subsets of stage I/II and stage III CRC samples by the resolution of 0.2. Furthermore, we also calculated the markers for each cluster of immune cell populations (Additional file 12: Table S7). The cell types of immune cells were defined according to the cell markers reported in a previous study [24]. We further verified the accuracy of T cell classification based on their pro-inflammatory and anti-inflammatory activity scores (Additional file 12: Table S8) using the AddModuleScore function.

### CNV inferring

We used the infercnvpy python package to obtain the somatic large-scale chromosomal copy number variation (CNV) score. Firstly, a log and normalized counts matrix of scRNA-seq data were processed, and the PCA (scanpy.pp.log1p) and neighbors finding (scanpy.pp.neighbors) were running by scanpy. Next, gene annotation and chromosome position files were prepared according to data requirements (https://github.com/broadinstitute/inferCNV). For each stage of the single cell data, we used infercnvpy.tl.infercnv to infer CNV and selected normal macrophages and fibroblasts as reference normal cells. The infercnvpy.pl.chromosome_heatmap and infercnvpy.tl.leiden were used to visualize the heatmap of CNV results and the Uniform Manifold Approximation and Projection (UMAP) of the reduction plot. Finally, the CNV scores were calculated by infercnvpy.tl.cnv_score and used to distinguish tumor cells from normal epithelial cells and immune cells (Additional file 13: Table S9).

### Pathway enrichment analysis

Pathway activity scores of single-cell data were calculated based on sc-TPA (https://github.com/zgyaru/testSctpa) software package according to a previous study [25]. The software scored each pathway activity after the integration of different score calculation methods. Pathway gene lists for enrichment analysis are downloadeded from MsigDB (https://www.gsea-msigdb.org/gsea/msigdb/). We mainly utilized the AUCell [26] function to calculate the pathway enrichment scores for different populations (Additional file 14: Tables S10–S11).

### Trajectory analysis

Monocle v.2 [27] (https://github.com/cole-trapnell-lab/monocle-release) was used to show the cell state transition in cancer cells for two CRC stages. We created a CellDataSet object based on UMI count matrices and the negbinomial.size parameter for the default setting. We got variable genes in the following cutoff criteria: dispersion_empirical > dispersion_fit; and mean expression > 0.1. In the trajectory analysis for tumor epithelial cells of the two stages, variable genes were substituted by epithelial differentiation marker genes for semisupervised trajectory reconstruction. DDRTree method and the orderCells function were used to reduce dimensional and order cells. Finally, we obtained the genes with significantly differential expression for pseudotime in different stages of tumor cells using differentialGeneTest function (Additional file 15: Table S12-13).

### Construction of EMT-invasion score for each cell

The intersection of stage I/II and stage III of pseudotime differential genes were used to analyze the survival time for TCGA-COADREAD data by survival package (*P* < 0.05, Additional file 16: Table S14). Further, we performed the Pearson test and selected the cutoff values of cor > (±) 0.1 and *P* < 0.05 as the pseudotime-associated genes (Additional file 16: Tables S15–S16). Next, we intersected the gene list of the two stages to obtain the EMT-invasion genes and their scores were then calculated using the AddModuleScore function.

### Re-localization for spatial transcriptome data

We utilized the SPOTlight [28] (https://marcelosua.github.io/SPOTlight/) package to relocate the single-cell data to the position spots of the spatial transcriptome data. Next, FindAllMarkers was used to get the marker genes for each cell type. Finally, we used the “spotlight_deconvolution” function to analyze 500 cells for each cell type, 2000 HVGs and nsNMF method to run the deconvolution step.

### Receptor ligand interaction analysis

To understand the communications between high EMT-invasion tumor cells and immune cells, we utilized the software Cellchat [29] (https://github.com/sqjin/CellChat) to calculate the receptor-ligand communication networks. CellChat is a computational tool designed to quantitatively infer and analyze cell–cell communication networks from single-cell RNA-sequencing data, enabling the prediction of major signaling interactions and their coordination in cellular functions. It achieves this by integrating prior knowledge of the interactions among signaling ligands, receptors, and their cofactors. Furthermore, we utilized the NicheNet [30] (https://github.com/saeyslab/nichenetr) to analyze the interaction intensity of the ligands and target genes between the upregulated and downregulated invasion genes. NicheNet is another computational tool that utilizes gene expression data from a method to predict ligand-target interactions among interacting cells based on their gene expression data with prior knowledge of signaling and gene regulatory networks. Actually, in addition to ligand-receptor interactions, NicheNet also incorporates intracellular signaling that sets NicheNet apart. In this analysis, we used NicheNet to pinpoint specific interacting cells and interaction pairs of interest, starting from specific gene sets.

### TCGA analysis

TCGA COAD and READ gene expression datasets of TPM and clinical datasets from Broad GDAC Firehose (https://gdac.broadinstitute.org/) were collected to obtain the mean of EMT gene signature. After downloading gene expression data from the Illumina HiSeq platform, we converted raw counts to normalized TPM values with log2-transformation. We selected COAD and READ samples with gene expression, stages, and overall survival clinical information. The top 50% of samples were classified as the group of the high-level scores, and the bottom 50% of samples as the group of the low-level scores. The ‘survival’ and ‘survdiff’ functions in R were used to generate Kaplan–Meier survival curves and calculate the *P* values of the log-rank test. The immune cell infiltration data was downloaded from TIMER [31] website (http://timer.comp-genomics.org/) for TCGA-COADREAD.

### Statistics

We used the Wilcoxon test to compare differences between two groups of data. Statistical significance among multiple cell types or sample types was determined using a one-way analysis of variance (ANOVA). The Pearson correlation test was used to correlate data with homogeneous variance. A *P*-value of > 0.05 indicates no statistically significant difference, and *P* ≤ 0.05, *P* ≤ 0.01, *P* ≤ 0.001, and *P* ≤ 0.0001 indicated varying degrees of statistically significance levels. All statistical tests were two-sided and performed using the R version 4.0.2.

## Results

### Overview of colorectal cancer TME characterized by scRNA-seq analysis

**Fig. 1:**
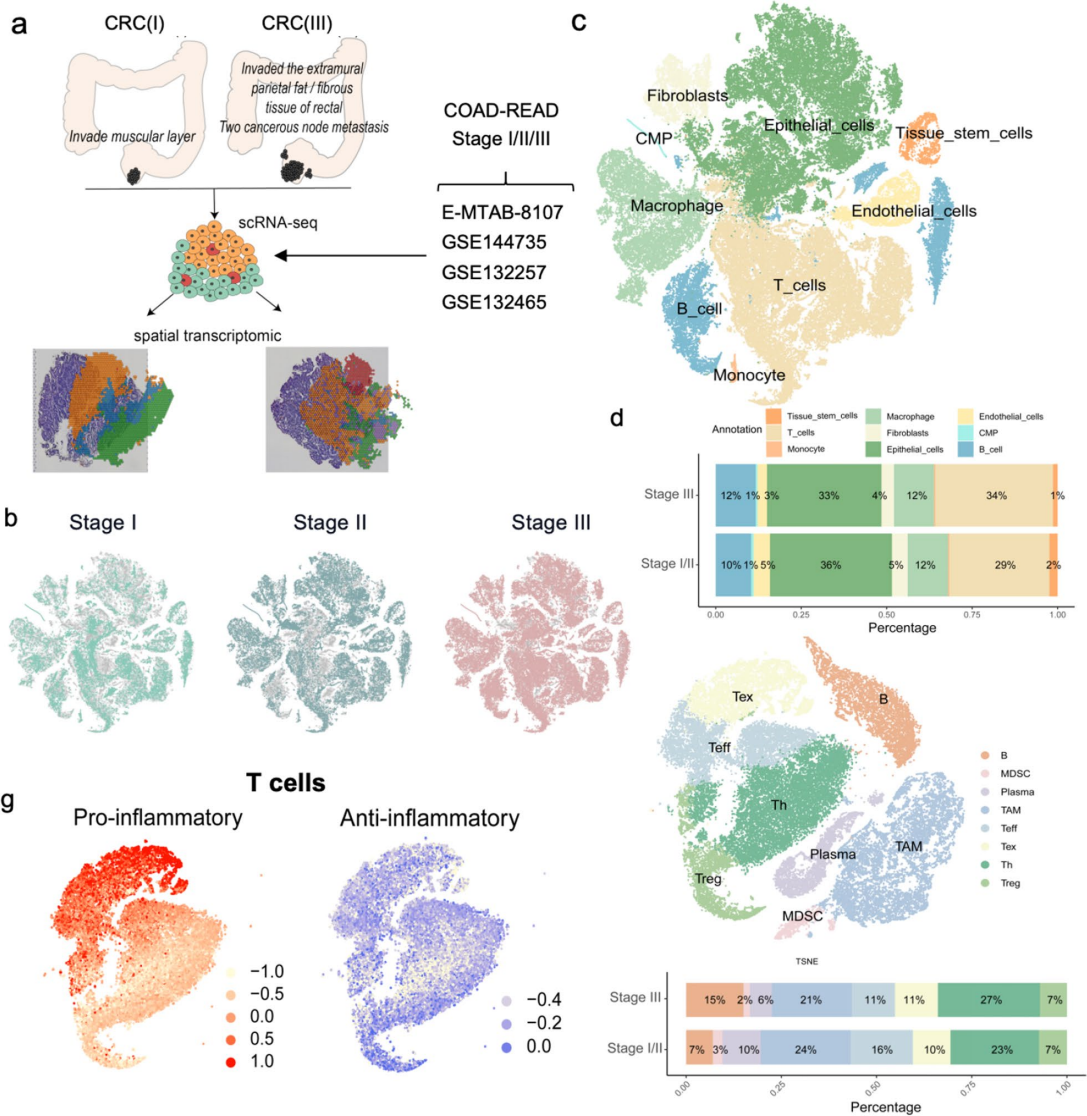
Single-cell gene profiling of colorectal cancer (CRC) tissues. **a** Illustration of sampling in this study. **b** tSNE plots of malignant and non-malignant cells colored by different stages of cancer samples **c**, tSNE plots for initial annotation on broader cell-type categories. CMP: common-myeloid progenitors. **d** The proportion of different cell types from the two stages of cancer samples. **e** tSNE plot showing the annotation of immune cell types for lymphocytes and myeloid cells. Th: T helper cells, TAM: tumor-associated macrophage, Teff: effector T cells, Tex: exhausted T cells, Treg: regulatory T cells. MDSC: myeloid-derived suppressor cells. **f** Proportions of immune cell types in stage I/II and stage III samples. **g** tSNE plots of T cells colored by pro-inflammatory (left) and anti-inflammatory (right) gene signatures

To decipher the cell composition within CRC, we performed scRNA-seq and spatial transcriptomics (ST) analyses on the two margin tissues of two CRC samples, one from stage I with invaded muscular layer and the other from stage III with metastasis into two cancerous nodes (Fig. 1a), using 10X Genomics. To increase the sample number of our single-cell data, we included additional four datasets obtained from the GEO and ArrayExpress databases, which were generated based on 49 samples of 42 CRC patients after removing the samples with distant metastasis or therapeutic treatment. The t-SNE plots indicate the position of cells for each dataset, the origin tissues and the patients within the atlas, as shown in Additional file 1: Figures S1a, b, and Additional file 1: Table S1–S3.

After strict quality control as described in Materials and Methods section, we selected 99,689 cells from the samples (*n* = 49), including 45,146 cells from stage I/II samples (n = 25) and 54,543 cells from stage III samples (*n* = 24), for further analysis (Additional file 12: Table S4, Fig. 1b). We performed clustering analysis (Additional file 1: Fig. S1d) and defined initial cell types using SingleR [22] and cell marker genes (Fig. 1f, Additional file 12: Table S5, Methods). We identified epithelial cells, T cells, macrophages, B cells, fibroblasts, tissue stem cells, common myeloid progenitors (CMP), and monocytes from the CRC samples (Fig. 1c). The epithelial cells exhibited high expression levels of epithelial markers, such as epithelial cell adhesion molecule (EPCAM), stratifin (SFN), and cytokeratins (KRT18/19) (Additional file 1: Fig. S1c). The proportion of immune cells in stage III was larger than that in stage I/II, with a ~ 5% increase in T cells (Fig. 1d), suggesting an activity increase of intratumoral immunity from stage I/II to stage III.

To identify the malignant tumor cells from all epithelial cells, we assessed the CNVs in the epithelial cell subgroups on a genome-wide scale (Additional file 2: Fig. S2a, b). After unsupervised clustering analysis, we derived CNVs from the multiple populations of epithelial and immune control cells for stage I/II (Additional file 2: Fig. S2c, left) and stage III (Additional file 2: Fig. S2d, left). As expected, the CNVs in most epithelial cell subsets were higher than those in macrophages and neutrophils. Furthermore, we observed that the CNV scores of specific epithelial cell clusters were markedly lower than that of other epithelial cells, which were subsequently removed and used as normal epithelial cells for both stages of CRC samples (Additional file 2: Fig. S2c, middle; Fig. S2c, middle). We retained the epithelial cells with higher CNV scores, indicating their properties of malignant epithelial cells, for further analysis (Additional file 2: Fig. S2d, right; Fig. S2d, right).

Moreover, we integrated SingleR and manual marker-based annotation to define immune cell subsets further for analysis. We identified eight main cell populations based on their marker genes (Fig. 1c, Additional file 3: Fig. S3a): T helper cells (Th, *N* = 12,416), tumor-associated macrophages (TAM, *N* = 10,838), effector T cells (Teff, *N* = 6605), exhausted T cells (Tex, *N* = 5241), regulatory T cells (Treg, *N* = 3531), B cells (*N* = 5720), plasma cells (*N* = 3714) and myeloid-derived suppressor cells (MDSC, *N* = 1009) from the CD45 + dataset. Notably, MDSCs were identified by high expression of CXCR1/2 (Additional file 3: Fig. S3b). The UMAP plot of T cells indicated that Tex and Teff are pro-inflammatory, while Treg and Th are anti-inflammatory in CRC, based on their gene expression signatures (Fig. 1g), which further validated the annotation results. Additionally, the analyses of myeloid cells, including TAMs and MDSCs, colored by inflammatory signatures, indicated that both TAMs and MDSCs are anti-inflammatory in CRC (Additional file 3: Fig. S3b). Notably, the proportion of Teff was relatively low and Th was high in stage III samples compared to those in stage I/II samples (Fig. 1f). Taken together with previous reports in multiple cancers (25–27), our data indicated that EMT is likely associated with the quantity of immunosuppressive T cells. In the following analysis, we further characterized the tumor TME in the EMT process of primary CRC at the temporal and spatial levels.

### EMT-associated tumor programs at the end of pseudotime

**Fig. 2:**
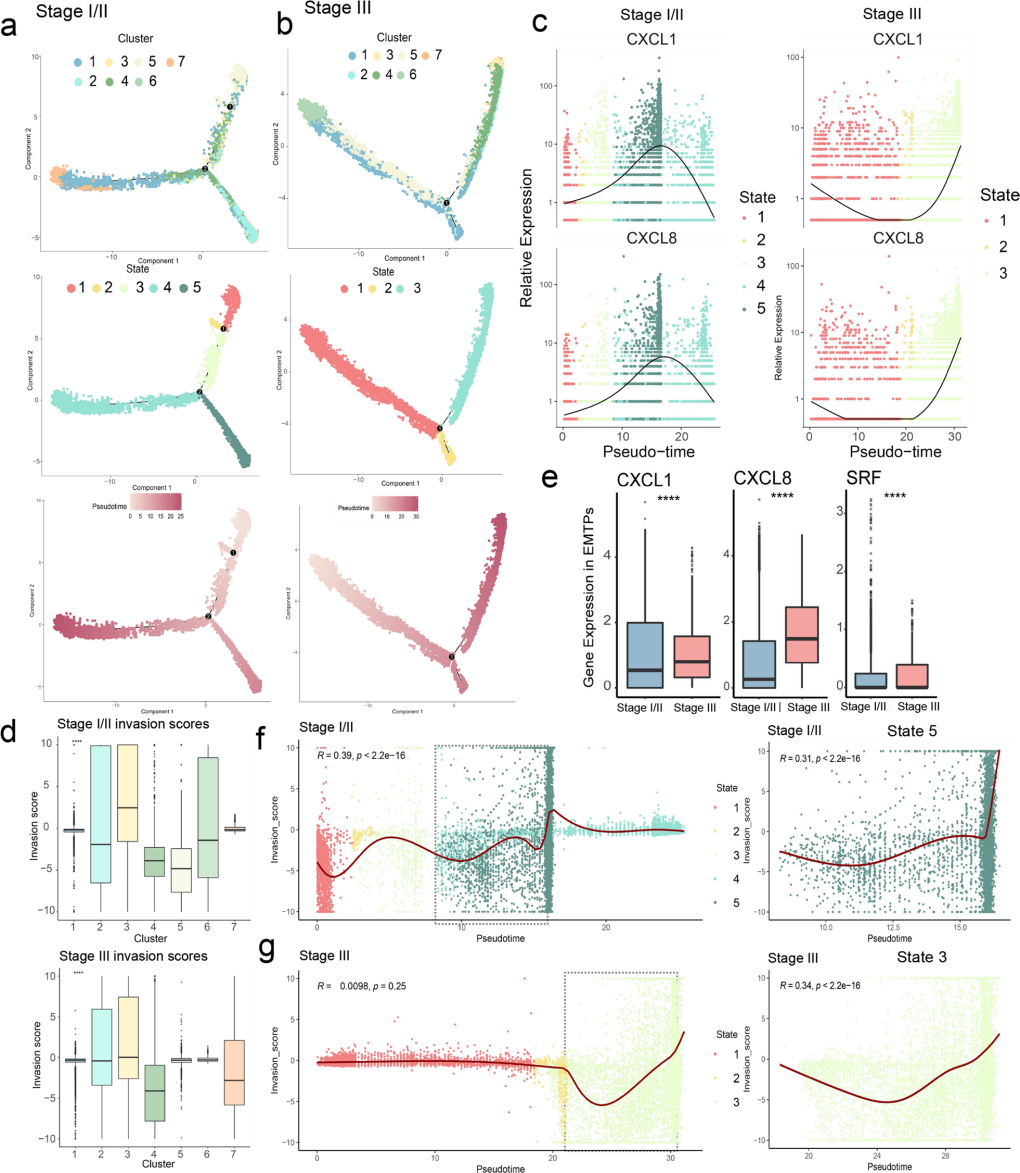
Malignant epithelial cells associated EMT-related tumor programs. **a** and **b** Pseudotime trajectory of CRC single cell transcriptomes, colored by cluster, pseudotime, and development state for stage I/II (**a**) and stage III (**b**) samples. **c** Kinetics plots showing relative expression of EMT invasion genes (CXCL1/CXCL8) across developmental pseudotime and development state for stage I/II and stage III samples. **d** and **e** Boxplots of gene expression analyses. The data show differential expression of the EMT invasion score across clusters (1–7) (**d**), and EMT invasion genes across EMTPs (**e**) for stages I/II and III samples. **f** Kinetics and scatterplots showing the positive correlation between the pseudotime and EMT invasion scores for stages I/II (**f**) and III (**g**) samples. Pearson correlation coefficients (R values) and *P* values are indicated. The square region indicates the cell plot for state 5/3 (right), and the independent correlation coefficients and P values for state 5 and state 3 are provided

Given the heterogeneous cellular compositions across different stages (Additional file 2: Fig. S2c, d), we asked whether and how EMT process varied with pseudotime among different malignant epithelial populations. Before addressing this, we utilized Monocle v.2 [27] to estimate the pseudotime of each cell, which used machine learning reverse graph embedding to order malignant cells with distinct cellular fates or biological processes. By reducing the dimensionality of the data, we identified 7 clusters and 5 states for stage I/II malignant cells and 7 clusters and 3 states for stage III malignant cells (Fig. 2a, b). Both clusters and states represented types of cell populations that focus on gene expression or pseudotime. Additionally, the trajectory inference analysis further identified a clear developmental trajectory from population state 1 to state 4 and state 1 to stage 5, as another trajectory for stage I/II malignant cells. Similarly, the developmental process from population state 1 to state 2 and state 1 to stage 3 was identified as another trajectory for stage III malignant cells (Fig. 2a, b). Our data revealed regulatory processes for cells and helped to identify cell’s differentiation endpoints for their pseudotime.

We observed an enrichment of MYC targets, oxidative phosphorylation, and the reactive oxygen species (ROS) pathway in state 5 of stage I/II samples and state 3 of stage III samples, based on the analysis shown in Additional file 4: Fig. S4. The biological function of cell populations at the endpoint of the trajectory likely promoted EMT of malignant cells in the processes of tumor proliferation, metabolism and migration, according to previous reports [32, 33]. Similarly, MDSCs enriched in the margin-invasive niches could also inhibit anti-tumor T cells through ROS production, manifesting the important role of the ROS pathway in the margin-invasive niche of CRC [34].

We hypothesized that some EMT invasion genes could be functionally increased with pseudotime in tumor cell populations. Therefore, we constructed a set of EMT invasion genes by collecting them from the differential gene set of PT-states, and filtered them based on significant prognostic values validated in TCGA samples (overall survival time, log-rank *P*-value < 0.05). We further obtained the intersected genes significantly correlated with pseudotime for both stage I/II and stage III tumor programs (see Materials and Methods section). Finally, we retained three valuable genes: CXCL1, CXCL8, and SRF that exhibited the highest expression in state 5 and state 3 populations at the endpoint of the evolutionary trajectory (Additional file 5: Fig. S5a). Notably, CXCL1 and CXCL8, which are the key functional genes in the hallmark pathway of EMT (Additional file 5: Fig. S5b), are essential for the activation and trafficking of inflammatory mediators as well as tumor progression and metastasis.

Along with the increased in pseudotime, the expression of CXCL1/CXCL8 is not linear in the two stages (Fig. 2c) due to the influence of different trajectories on gene expression tendency, while SRF showed no significant change of pseudotime (Additional file 5: Fig. S5d). We then constructed an EMT-invasion score based on the three EMT-pseudotime genes for the two CRC stages using Seurat (Additional file 5: Fig. S5e). Notably, the scores in cluster 3 for both stages were the highest and located at the endpoint of the pseudotime evolutionary trajectory (Fig. 2d, Additional file 5: Fig. S5f, ANOVA test, *p* < 0.0001). Given that the change in EMT-invasion score does not show a clear tendency for all state populations (Fig. 2f–g, left, Additional file 4: Fig. S4e), we selected the individual state population for correlation analysis. It is reasonable to observe that the pseudotime in state 5 (Fig. 2f, right, R = 0.31, *P* < 2.2e-16) and state 4 (Additional file 5: Fig. S5g, R = 0.46, *P* < 2.2e-16) for stage I/II and state 3 (Fig. 2g, right, R = 0.34, *P* < 2.2e-16) for stage III samples showed a significant correlation with the EMT-invasion score, which was located at the endpoint of trajectory. The results indicate that these pseudotime-endpoint cell populations in each stage had relatively strong invasion ability compared to other populations (Fig. 2d). Notably, we have named these populations as pseudotime-endpoint EMT-invasion tumor programs (EMPTs), and the EMT invasion genes were highly expressed in the EMPTs of stage III compared to stage I/II samples (Fig. 2e, Wilcox test, *p*-value < 0.0001). In summary, the EMT-invasion genes can mediate a functional shift of EMT, which reflects the strength of tumor invasion and the direction of tumor development in CRC.

### Spatiotemporal relationship of tumor programs with EMT-status

**Fig. 3:**
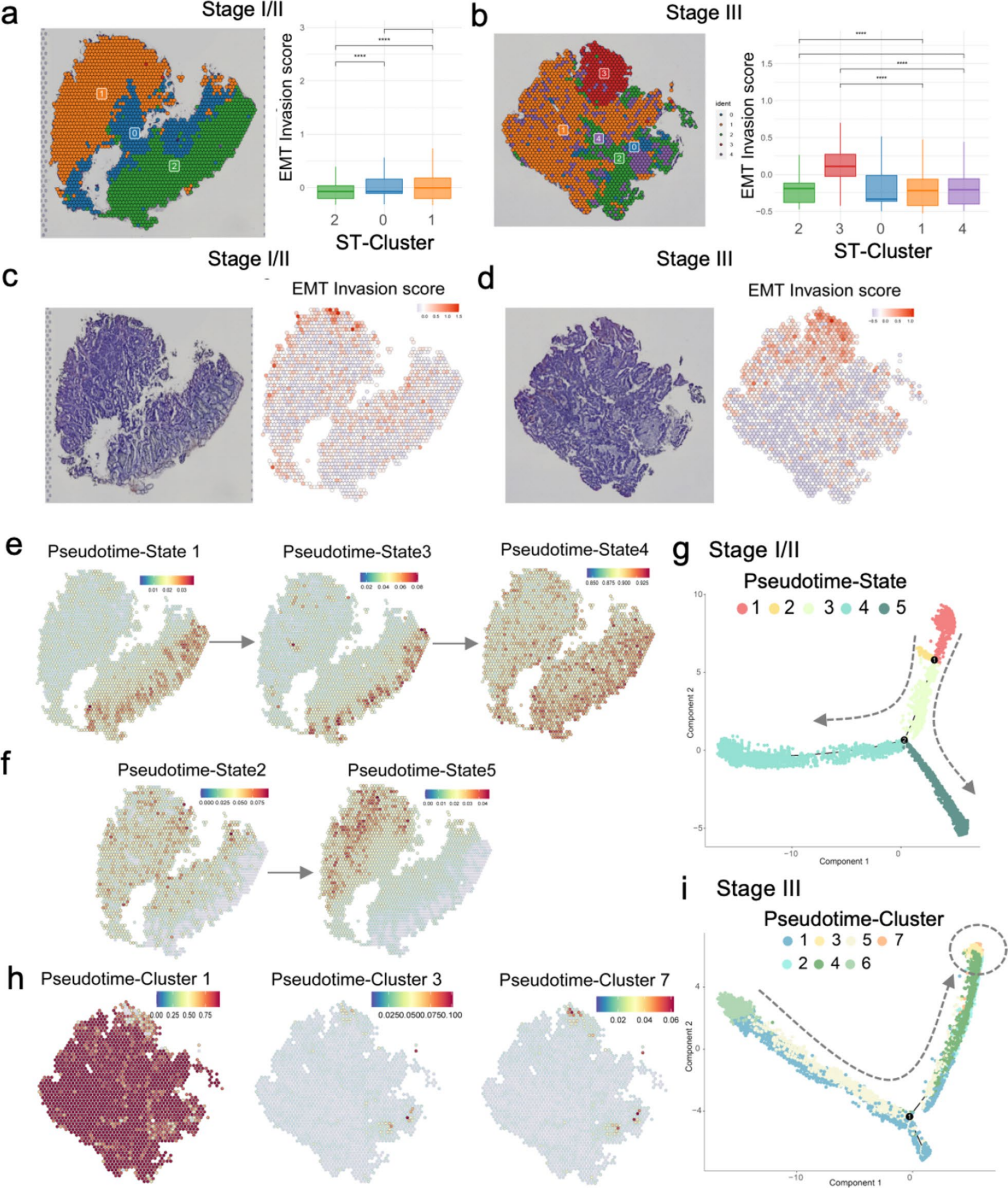
Spatiotemporal relationship of tumor programs with the EMT-status. **a** and** b** Distribution of the clustered spatial transcriptome data in hematoxylin–eosin (HE) stained images of the CRC samples of stages I (**a**) and III (**b**). Boxplots (right) shows the relative heterogeneity levels of EMT invasion scores across clusters.** c** and** d** HE-stained images (left) and distribution of EMT invasion scores across all spots (right) for stage I (**c**) and III (**d**) samples. **f** The cells of pseudotime-states 1, 3 and 4 likely belonging to the same evolutionary trajectory shown on spatial transcriptome data spots for stage I samples. **g** The cells of pseudotime-states 2 and 5 likely belonging to the same evolutionary trajectory shown on spatial transcriptome data spots for stage I samples. **h** The pseudotime trajectory colored by the development pseudotime-state for stage I samples. The dotted lines mean different evolutionary trajectories. **g** The cells of pseudotime-clusters 1, 3 and 7 likely belonging to the same evolutionary trajectory shown on spatial transcriptome data spots for stage III samples. **h** The pseudotime trajectory colored by pseudotime-clusters for stage III samples. The dotted line means the evolutionary trajectory, and the circle depicts the end of the evolutionary trajectory

In our further spatial analysis, we performed ST sequencing and clustering analysis on the two CRC samples. After quality control, we used 4,895 spots from the stage I sample, and 4,900 spots from the stage III sample for further analysis. Unsupervised clustering divided the two samples into distinct clusters (ST-clusters) (Fig. 3a, b, left) that were mapped to different regions on HE-stained images (Fig. 3c, d, left). We further observed significant enrichment of the EMT-invasion scores in ST-cluster 1, located in the margin of the right for stage I (Fig. 3c, right). In stage III, ST-cluster 3, located in the margin of the top region, showed significant enrichment of the EMT-invasion score (Fig. 3c, right). Moreover, the margin of the right region, including ST-clusters 2 and 4, showed significant enrichment of the EMT-invasion scores to some extent.

To fully characterize the ST data, we mapped the staged paired single-cell transcriptome data to the ST data and located the PT (pseudotime) states of tumor population to the spatial position of HE-stained images. In stage I, the first trajectory of PT-states 1, 3 to 4 were mainly distributed to the right side of tumor lesion, and PT-state 4 was distributed to the right margin (Fig. 3e, g). The second trajectory of PT-states 2 to 5 was mainly distributed to the left side of tumor lesion, and PT-state 5 was distributed to the left margin (Fig. 3f, g). In addition, the results of PT-cluster, a supplement cluster for PT-state, indicated that PT-clusters 7 and 2 located at the trajectory endpoint were mainly distributed to the right but not the left margin for an unknown reason (Additional file 6: Fig. S6a, b). In stage III, the trajectory for PT-clusters 1 to 3 was mainly distributed to the right side of tumor lesion, and PT-cluster 7 was mainly distributed to the upside of the tumor lesion (Fig. 3h, i). However, for unknown reasons, the results of PT-states were not clear enough to support this conclusion (Additional file 6: Fig. S6c, d).

Among these tumor clusters, we defined the PT-states 4/5 in stage I/II samples and PT-clusters 3/7 in stage III samples as the pseudotime-endpoint EMT-invasion tumor programs, or EMTPs, with increased expression of EMT invasion genes and located at the endpoint of development, which are most likely the forward for tumor invasion. These EMTPs are likely distributed to the growing margin of tumor lesion space. Also, in our additional ST datasets of CRC, we noted a significant enrichment in invasion scores along the tumor region's periphery (Additional file 7: Fig. S7). The results highlighted the spatiotemporal dynamic tumor cell landscape of CRC patients across tumor invasion process.

### Quantification of immune cell diversity adjacent to the EMTPs

**Fig. 4:**
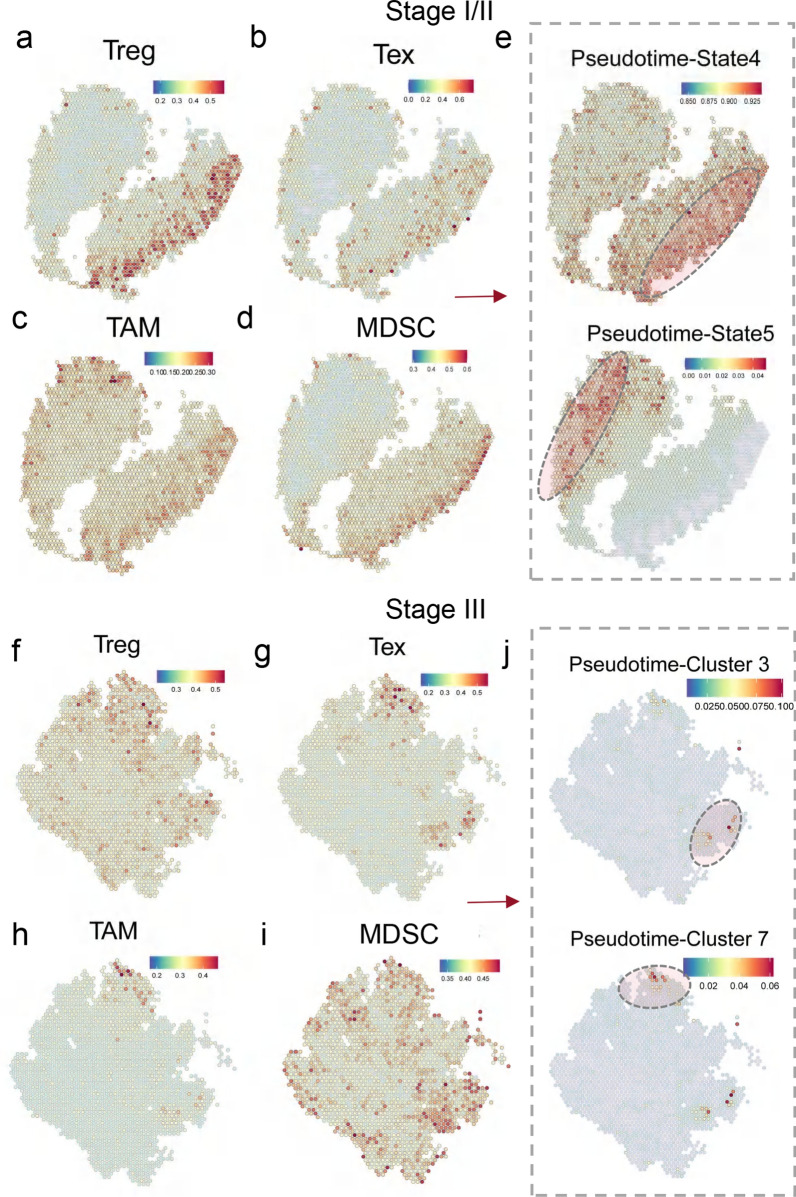
Quantification of immune cell diversity adjacent to the high-EMT tumor programs.** a**, **b**, **c** and **d** Co-localization of Treg (**a**), Tex (**b**), TAM (**c**), and MDSC (**d**) cells in stage I samples using the spatial transcriptome data. **e** Co-localization of the cells of states 4 (up) and 5 (down) for stage I malignant tumor cells at the end of evolutionary trajectory. **f**, **g**, **h** and **i** Co-localization of Treg (**f**), Tex (**g**), TAM (**h**), and MDSC (i) cells in the stage III samples determined by the spatial transcriptome data. **e** Co-localization of the cells of clusters 4 (up) and 7 (down) for stage III malignant tumor cells at the end of evolutionary trajectory

To better understand the EMTPs that may drive the interactions with the immune system, we next sought to identify the patterns of spatially adjacent immune cell types across EMTPs. We mapped all immune cells derived from different biopsies of stages using single-cell expression data to focus on the overlapped spatial location of EMTPs. Across ST of stage I, four types of immune cells, including Treg, Tex, TAM and MDSC, were found to be distributed to the same location as PT-states 4 and 5 (Fig. 4a–d). The locations of Treg and MDSC specifically overlapped with PT-state 4, while TAM overlapped with both endpoint PT-states 4 and 5 (Fig. 4e), including both directions of evolution of stage I/II. Similarly, these immune cell types were also distributed to the same location as PT-clusters 3 and 7 in the stage III biopsies, which included two cancerous nodes of metastases (Fig. 4f–i). The infiltration levels were also significantly elevated in the high level of EMT invasion scores for Treg, TAM and MDSC in bulk cancer samples of TCGA (Additional file 7: Fig. S7a, Wilcox test) obtained from the TIMER website. Other immune cells, such as Teff and plasma, were mainly enriched in stage I samples but not those of stage III (Additional file 8: Fig. S8b, c). Conversely, Th and B cells were mainly enriched in stage III samples but not in stage I (Additional file 8: Fig. S8b, c). However, all the locations of these immune cells did not clearly overlap with the region for EMTPs. Furthermore, by examining the co-localization of immune cells in an additional set of three spatial sample datasets, we clearly observed significant enrichment of myeloid lineage cell types, including TAM and MDSC, in these tumor samples (Additional file 9: Fig. S9).

Collectively, our observation of distinct EMTPs spatially overlapping with different immune cells may explain previously reported phenomena of intimate interaction between heterogeneously infiltrated malignant cells and infiltrated immunosuppression cells in pre-metastasis [16, 35, 36]. In fact, the presence of these EMTP adjacent immunosuppressive cells strongly suggest their causative role of EMTP invasion in the pre-metastasis niche of CRC [37, 38].

### Interaction of EMTPs with immune cells

**Fig. 5:**
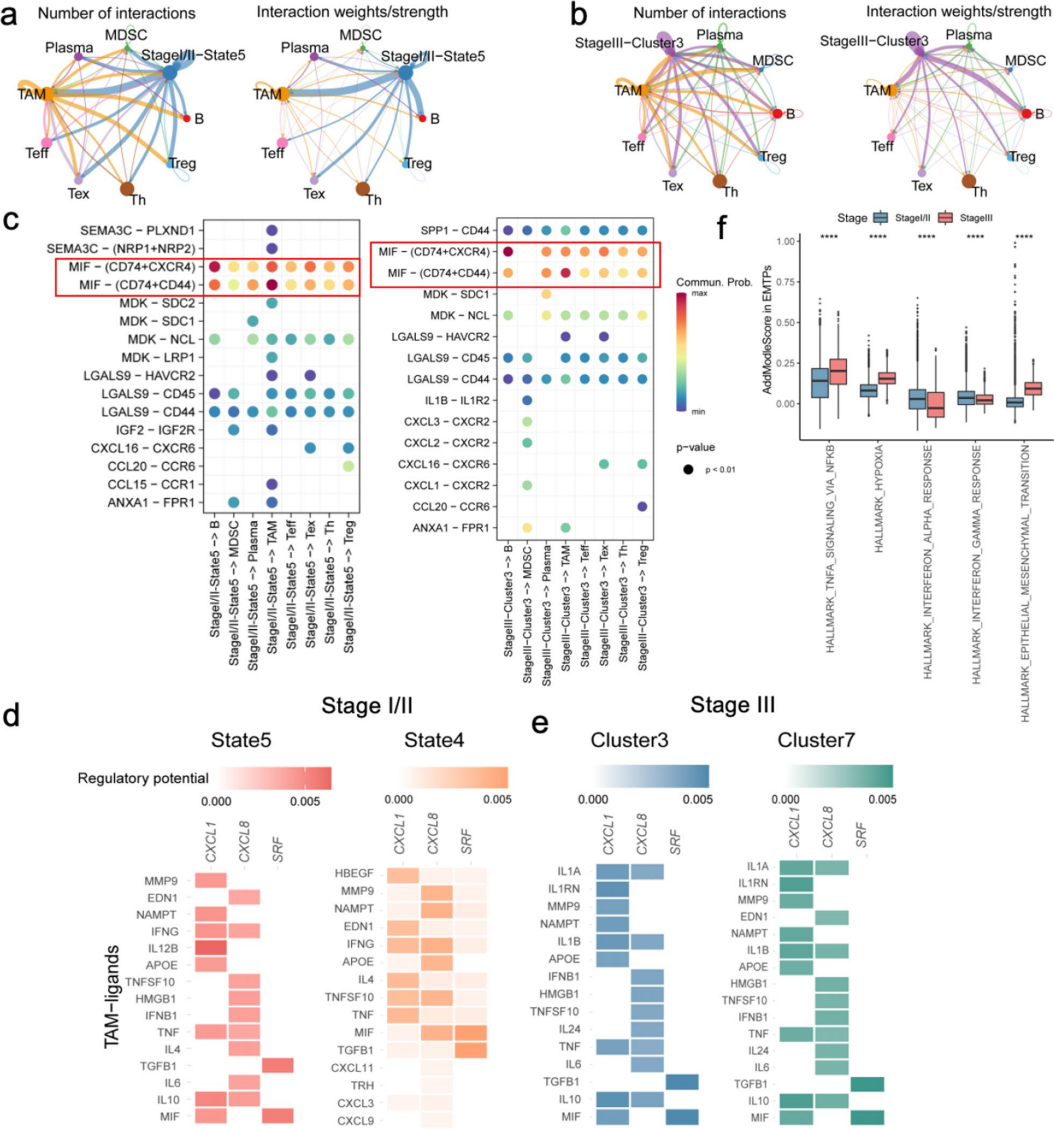
Interactions of higher EMT tumor programs with immune cells. **a** and **b** The number and interaction strength of the receptor-ligand interaction among the state 5 (**a**) and cluster 3 (**b**) tumor programs and other immune cells in stage I/II and III samples. Line thickness is proportional to interaction numbers (left) and strength (right). **c** Probability of receptor-ligand interactions with immune cells for the EMTPs of state5 in stage I/II samples and EMTPs of cluster3 in stage III samples. **d** The heatmaps of the ligand-target gene interactions between the states 5 (left)/4 (right) and TAM of the three target EMT invasion genes in stage I/II samples. **e** The heatmaps of the ligand-target gene interactions between the clusters 3 (left) and 7 (right) programs and TAM for the three EMT invasion genes in stage III samples.** f** Boxplot for the difference of hallmark pathways in different EMTPs of the two stages. The hallmark pathway scores were calculated by the AddModuleScore function and Wilcox test

Given the observation of TAM, MDSC, Treg, and Tex adjacent to the EMTPs in the CRC samples of for both stage I and III, we hypothesized that EMTPs participated in a complex crosstalk with tumor-associated immune cells. In this study, we extensively assessed the interaction of EMTPs with adjacent immune cells via known receptor-ligand pairs using CellChat [29] (Fig. 5a, b). Then we utilized NicheNet [30] to infer the ligand-target interaction of EMT invasion genes for precise dissection. Based on the observation of the number and strength of interactions for EMTPs and PT-state 5 (Fig. 5a) in stage I/II and PT-cluster 3 in stage III (Additional file 10: Fig. S10a) with other immune cells, we found that both EMTPs in both cases were inferred to signal to TAM for the expression of macrophage migration inhibitory factor (MIF), which interacted with CD74, CD44, and CXCR4 (Fig. 5c). Among them, CD74 plays a pivotal role in maintaining tumor homeostasis by releasing a tumor escape signal to inhibit T cell activity [39]. CD44 is a non-kinase transmembrane glycoprotein that is highly expressed in metastasized tumors, while CD44 variants may play a role in the EMT and adaptive plasticity of cancer cells [40]. Meanwhile, CXCR4, the most well-studied chemokine receptor, has a role in regulating cell progression and metastasis [41]. Similarly, EMTPs for both PT-state 4 in stage I/II and PT-cluster 7 in stage III were also enriched in the interaction of MIF-(CD74 + CD44) with TAM and other immune cells (Additional file 10: Fig. S10a, b and c).

We also observed evidence of interactions between EMTPs and EMT invasion genes. Consistent with the results of CellChat, the MIF gene was also inferred by ligand-target genes interaction as ligand genes upon NicheNet (Fig. 5d). MIF was previously found to recruit TAM to liver pre-metastatic niches and induce fibronectin via TGF in pancreatic cancer [42]. Furthermore, the results for TAM showed that MIF only interacted with CXCL8 for PT-state 4 in stage I/II but not stage III (Fig. 5d, e). Consistent with TAM, other regulatory and suppressive immune cells, such as MDSC, Treg and Tex, showed no interaction with MIF-CXCL8 in stage III samples (Additional file 11: Fig. S11). A critical function of CXCL8 in tumors is the activation and trafficking of inflammatory mediators, promoting tumor growth and metastasis [43, 44]. From the results above, Teff was found to be more enriched in stage I spatial data than that in stage III samples (Additional file 8: Fig. S8a, b). Taken together, these results indicated that the presence of metastasis niches of stage III is different from stage I in CRC.

On the other hand, another type of EMTP-adjacent immune cells, Tex, likely used interferon signaling to cause cancer cells and immune cells to negate each other and establish a regulatory relationship that attenuates both adaptive and innate immune killing [45, 46]. IFNG, produced by Tex (Additional file 9: Fig. S9a, b), also exhibited increased interaction with CXCL8/1 in the EMTPs of both stages, which is critical for CRC tumor invasion and deserves further analysis.

Strikingly, the difference analysis of hallmark pathways indicates that the EMTPs are enriched in NF-κB and hypoxia signaling for stage III samples, and interferon α/γ immune response for stage I samples (Fig. 5f, Wilcox test, *P*-value < 0.0001). Combining the previous results, we conclude that the margin invasive niche of stage III samples is hypoxic, which is different from the inflammatory niche of stage I in CRC samples. In summary, tumor cell-derived chemokines and cytokines, such as CXCL1/CXCL8 and SRF, recruit MDSCs, TAMs, Treg and Tex, and interact with target genes, like MIF and IFNG. These recruited regulatory and suppressive immune cells enhance metastasis through promoting the formation of margin invasive niches.

## Discussion

In the current study, we characterized the cellular and spatial tumor and immune landscape of tumor margin invasive niches in primary CRC using the state-of-the-art high-throughput scRNA-seq and ST for stage I/II and stage III samples. We defined the malignant cell population with pseudotime states and clusters as EMTPs with high EMT ability and at the endpoint of pseudotime trajectory (Figs. 2a, b; 3). These EMTPs were enriched in the hallmark pathways of MYC-target, oxidative phosphorylation, and ROS pathways, which play central roles in almost every aspect of oncogenic process, orchestrating cell proliferation, apoptosis, differentiation, metabolism and immune surveillance [47, 48]. Interestingly, both stages of EMTPs were found at the edge of tumor lesions and possessed higher expression levels of EMT-invasion genes than interior tumor cells. Consistently, this phenomenon was also reported in other cancers, in which tumors generally grow from the cores to the invasion edges [49, 50]. In summary, EMTPs are important causal factors for the margin invasive niches.

By quantifying immune cells of scRNA-seq data and ST data at the locations of HE-stained images, we observed that suppressive TAM, MDSC, Treg and Tex were located in the regions spatially adjacent to EMTPs, suggesting that intratumor immunosuppressive cells were highly active in invasion edge (Fig. 4). Interestingly, the distribution of immunosuppressive cells did not change significantly between the two tumor stages, and they were reported to have a crucial role in metastatic CRC [51]. However, in stage I samples, a large fraction of Teff and a small fraction of Th and B cells were identified, while an opposite trend was observed in stage III samples. As for the underlying mechanism, one possibility is that such immune reprogramming in stage I samples was induced by an inflammatory margin invasive niche to help tumor growth and break through the basal layer, which was also reported in other studies [52, 53]. Therefore, the pro-inflammatory mediator IFNG secreted by stage I/II margin invasive niches induced chemokines CXCL1 and CXCL8 associated with TAM activation. Previous analyses have reported that CXCL8 expression was negatively correlated with Erα expression and linked to increased invasiveness potential of breast cancer [54]. CXCL1 has also been shown to promote the progression of tumors and participate in the angiogenesis of colon cancer and melanoma [55, 56].

In general, MIF secreted by EMTPs in both stages had high interactions with ligand CXCL4/CD74/CD44 and target genes CXCL1/SRF, inducing the recruitment of immune cells, especially TAMs (Fig. 5c, d). However, in stage III samples, we observed that increased interactions between MDSCs and CXCL1/2/3 chemokines, and EMTPs expressed higher levels of EMT-invasion genes than stage I samples, indicating a different and progressive process. Pathway analysis verified the hypoxic margin invasive niche in stage III CRC. Previous studies also reported that the presence of MDSCs led to elevated ROS production and increased immunosuppression, augmenting metastasis [57]. Moreover, MDSC-mediated immunosuppression supported by TNF signaling in liver pre-metastatic niche formation can promote liver metastasis [58]. The above results indicate that both pre-metastatic niche and invasive niche of early-stage tumors are characterized by the formation of an immune-suppressive TME, facilitating the ingress of tumor cells.

The immune cells residing at tumor's edges play multifaceted roles. They can serve as either accomplices or adversaries of the tumors [59, 60]. This prompts us to consider the possibility of intervening with pharmacological agents at the expanding tumor edges, aiming to transform these barrier cells into supportive entities. Such an intervention may hold the potential to impede tumor dissemination and hinder their growth. Indeed, the interaction between the EMT-invasion CXCL8 with its receptors CXCR1 represents a promising therapeutic target, as demonstrated by multiple ongoing clinical trials [61–65].

Our studies bear several limitations and unresolved issues. Firstly, we only examined the outcomes of a limited number of CRC samples, with a limited number of spatial samples as well. This raised the question of whether a more extensive dataset would reveal additional insights. Secondly, if the microenvironment at tumor expansion periphery exhibits similarity to pre-metastatic microenvironment, can we extend this phenomenon to other cancer types? This intriguing query deserves further investigation. Furthermore, it is imperative to underscore that the results of our analysis need to be validated by wet-lab molecular biology experiments which is a critical gap in our study. To complement this gap, we reviewed a number of previous studies that used molecular experimental approaches to explore the inhibitory effects of immune cells within TME [66–70]. These studies focused on the factors, CXCL1, CXCL8 and SRF, which exhibited high immune suppression potential and promote tumor cell migration and invasion. These effects have been observed in various cancer types, including glioblastoma [70], breast cancer [71, 72], and gastric cancer [73, 74] to impact the overall survivals of cancer patients. The results in these previous reports are consistent with the crucial roles of the invasion genes in the progression of CRC progression identified in this study.

In conclusion, our current study reveals a previously unappreciated spatiotemporal immune landscape of EMTPs in primary CRC, and provides the spatiotemporal characteristics of the pre-invasive niche for different stages of primary CRC.

### Supplementary Information

**Additional file 1:** Collected datasets.

**Additional file 2:** Patients cell number statistics.

**Additional file 3:** Samples cell number statistics.

**Additional file 4:** Clinical stage cell number statistics.

**Additional file 5:** Cluster cell number statistics.

**Additional file 6:** Marker genes for first annotation.

**Additional file 7:** Marker genes for immune cells.

**Additional file 8:** Marker genes for T cells state.

**Additional file 9:** InferCNV results for Epithelial cells.

**Additional file 10:** Top 5 enriched pathways for Epithelial clusters in stage I/II.

**Additional file 11:** Top 5 enriched pathways for Epithelial clusters in stage III.

**Additional file 12:** Stage I/II Differential Genes.

**Additional file 13:** Stage III Differential Genes.

**Additional file 14:** Survial analysis for pseudotime differential genes.

**Additional file 15:** Correlation for genes and Pseudotime in stage I/II.

**Additional file 16:** Correlation for genes and Pseudotime in stage III.

## Data Availability

The published scRNA-seq and metadata collected for the SMC and KUL3 cohorts are available in the NCBI Gene Expression Omnibus (GEO) database under the accession codes GSE132465 [75], GSE132257 [75] and GSE144735 [75]. E-MTAB-8107 [76]. The TCGA RNA-seq data and survival data were downloaded from GDAC Firehose (https://gdac.broadinstitute.org). The datasets for this study are available from https://data.mendeley.com/drafts/mdr6mgmvz3. All code to reproduce this study is publicly available on GitHub (https://github.com/dengchunyu/STrectal).
