# Supplementary figures and images for: Unraveling temporal and spatial biomarkers of epithelial-mesenchymal transition in colorectal cancer: insights into the crucial role of immunosuppressive cells
Source: J Transl Med. 2023 Nov 8;21:794. doi: 10.1186/s12967-023-04600-x (PMC10633927; doi:10.1186/s12967-023-04600-x)

**a** Two CRC samples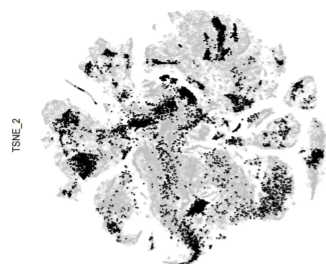

E-MTAB-8107

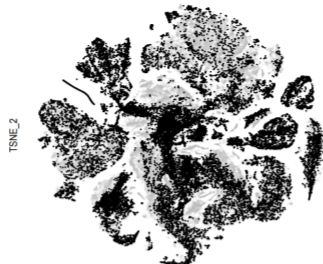

GSE132465

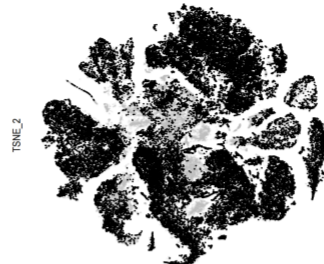

GSE144735

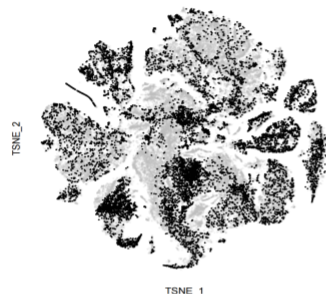

GSE132257

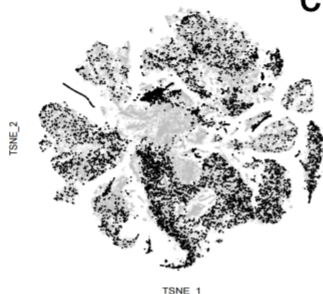**c**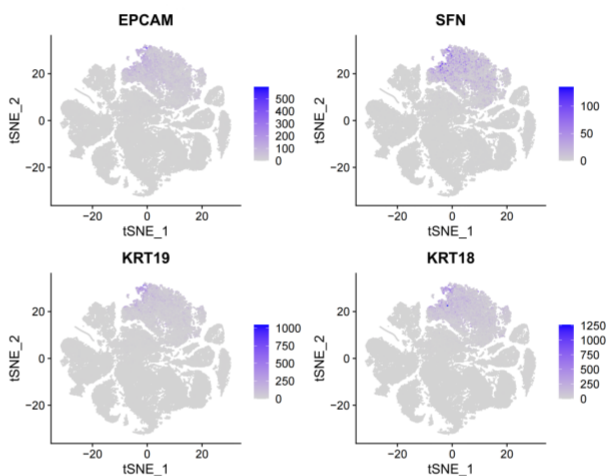**b** Samples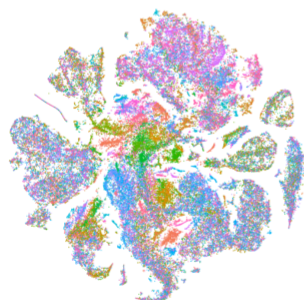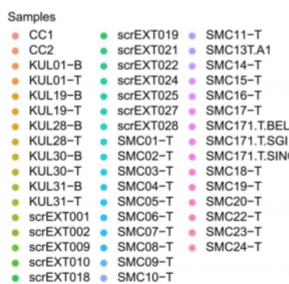

Patients

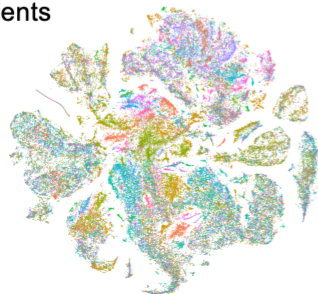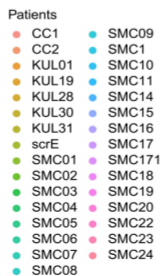**d**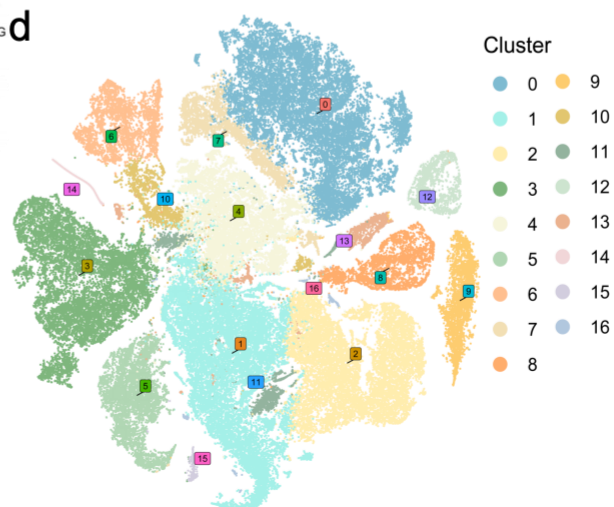

Cluster

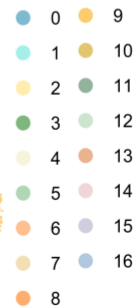

Supplement: Supplementary file 1 — Additional file 1: Collected datasets. [file 12967_2023_4600_MOESM1_ESM.pdf]

## Stage I/II

a

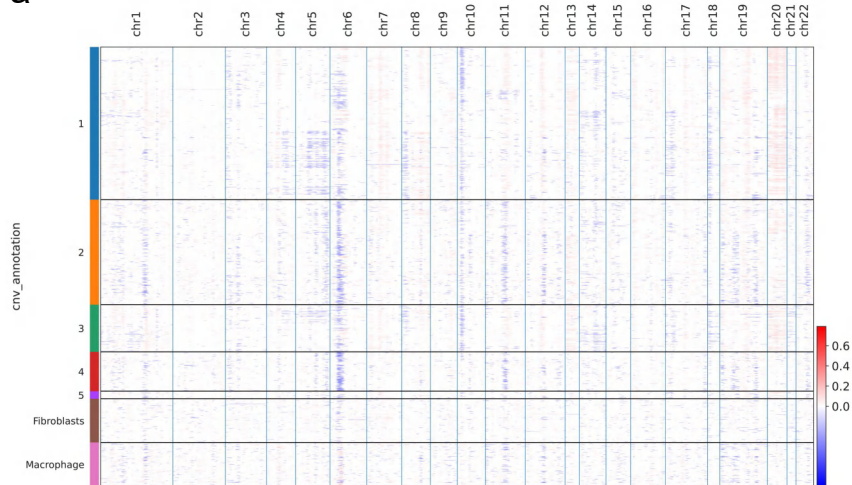

b

## Stage III

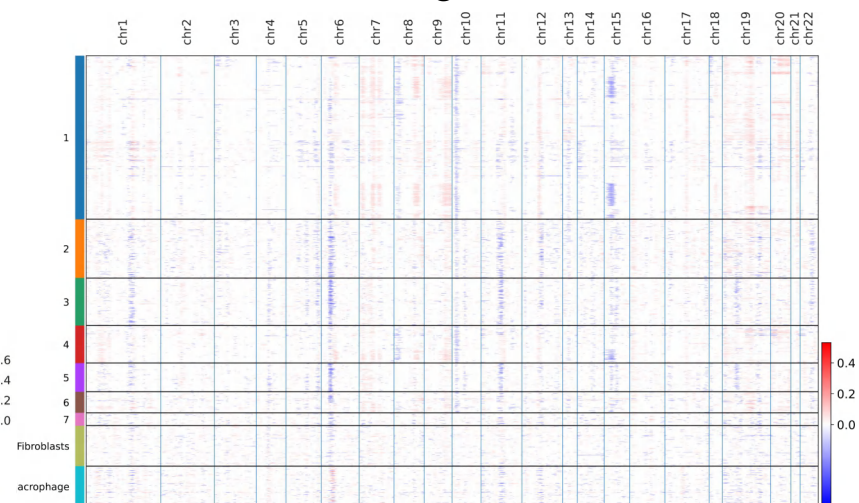

## Stage I/II epithelial cell

c

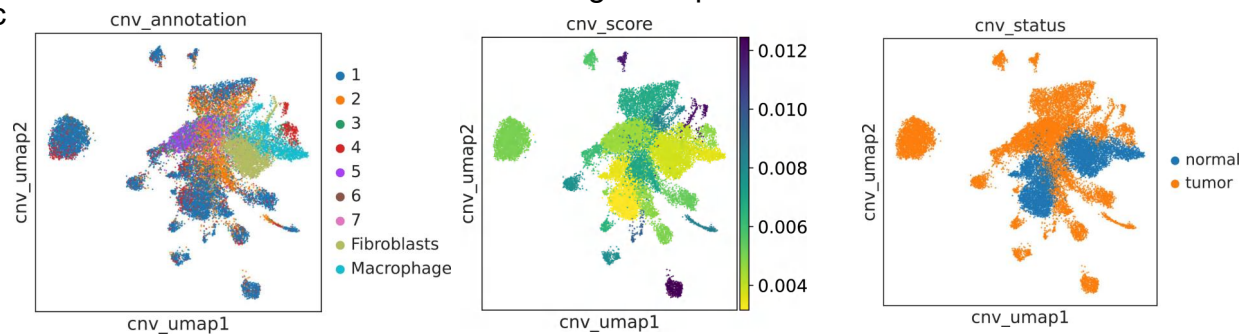

## Stage III epithelial cell

d

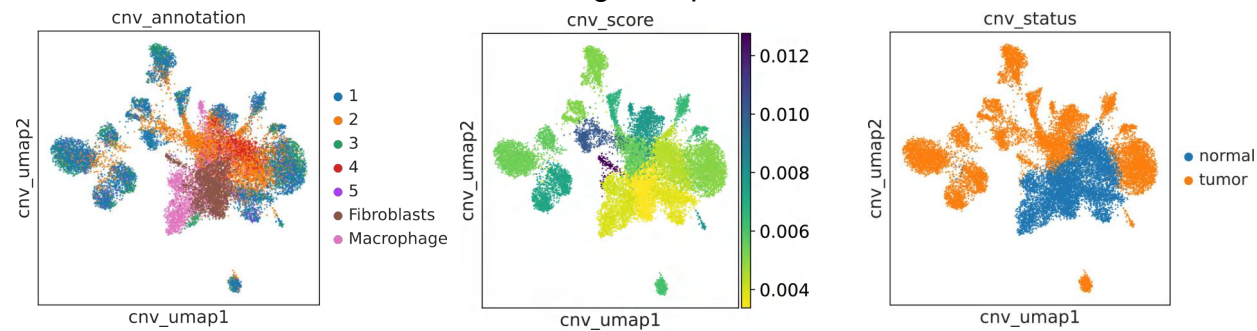

Supplement: Supplementary file 2 — Additional file 2: Patients cell number statistics. [file 12967_2023_4600_MOESM2_ESM.pdf]

**a**

Identity

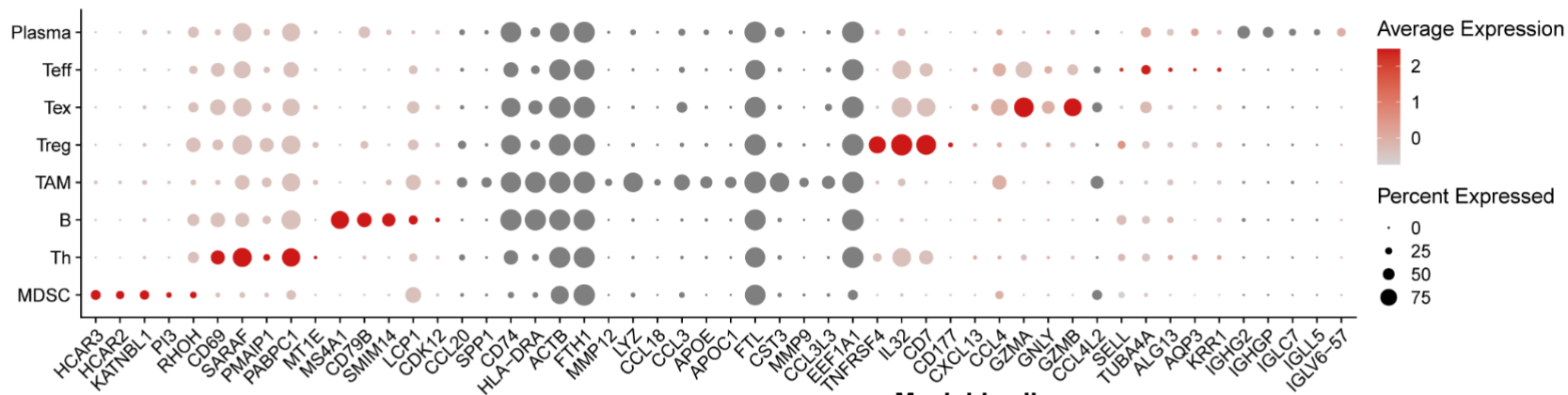**b**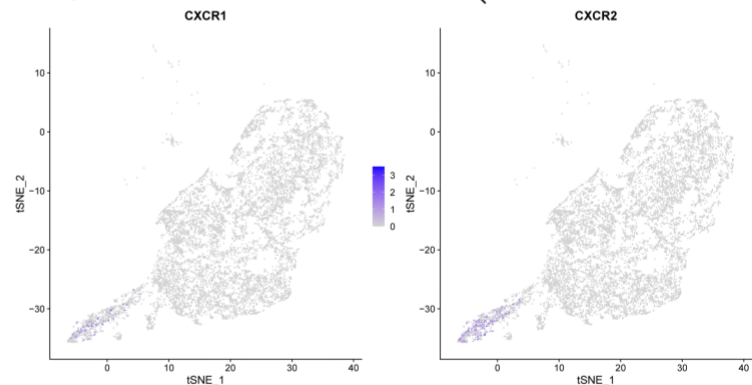**c**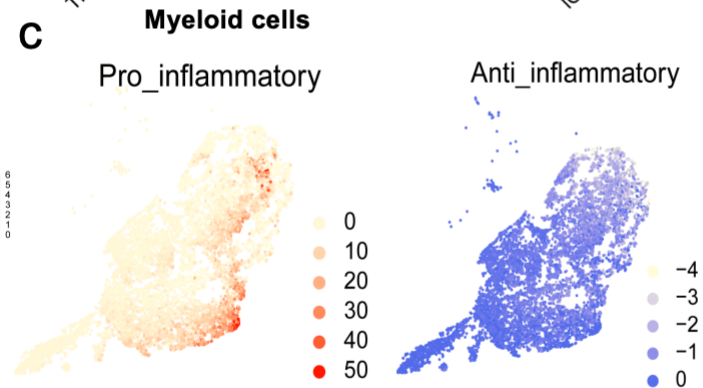

Supplement: Supplementary file 3 — Additional file 3: Samples cell number statistics. [file 12967_2023_4600_MOESM3_ESM.pdf]

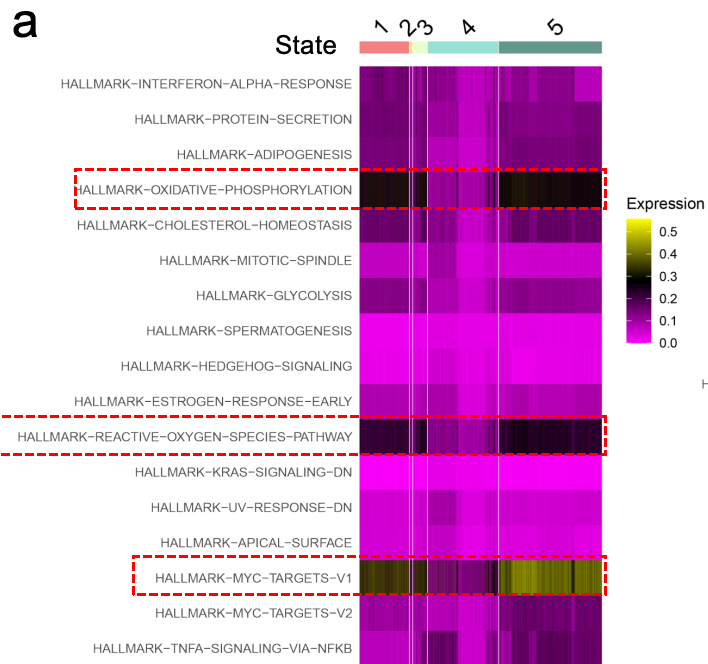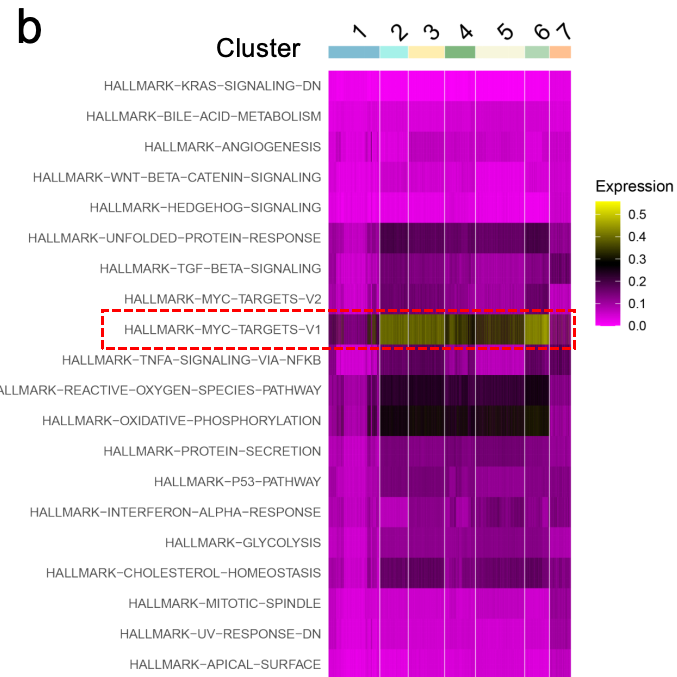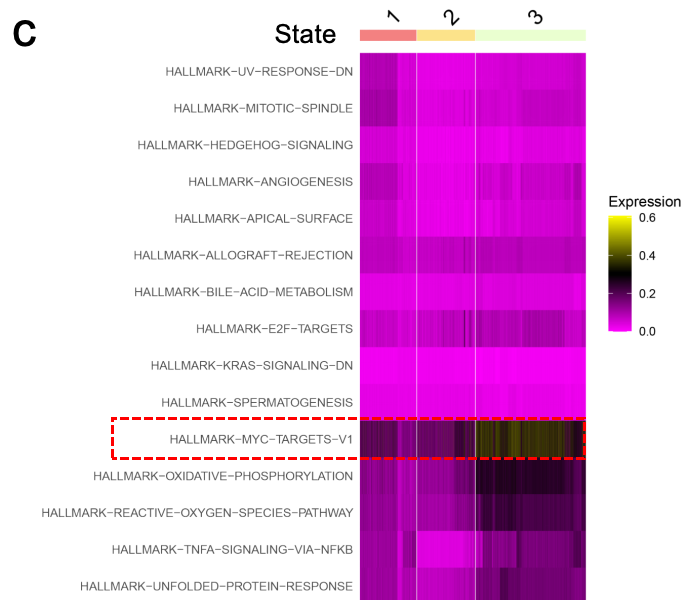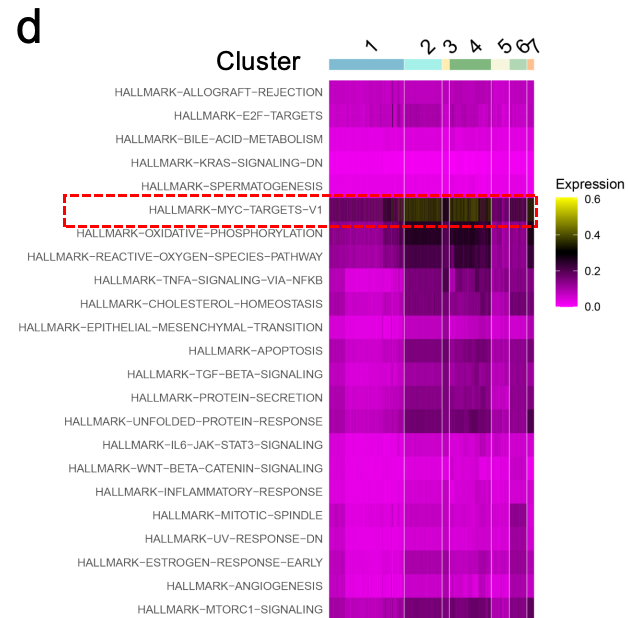

Supplement: Supplementary file 4 — Additional file 4: Clinical stage cell number statistics. [file 12967_2023_4600_MOESM4_ESM.pdf]

**a**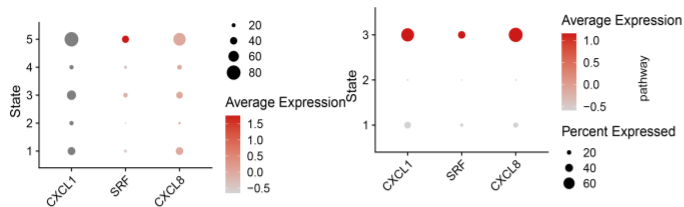**b**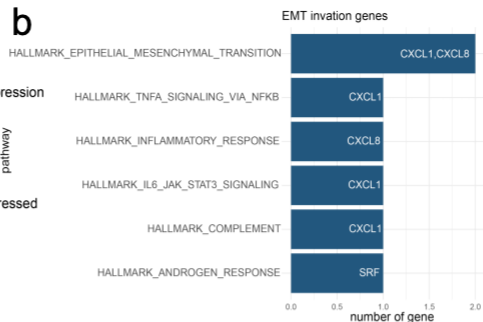**c**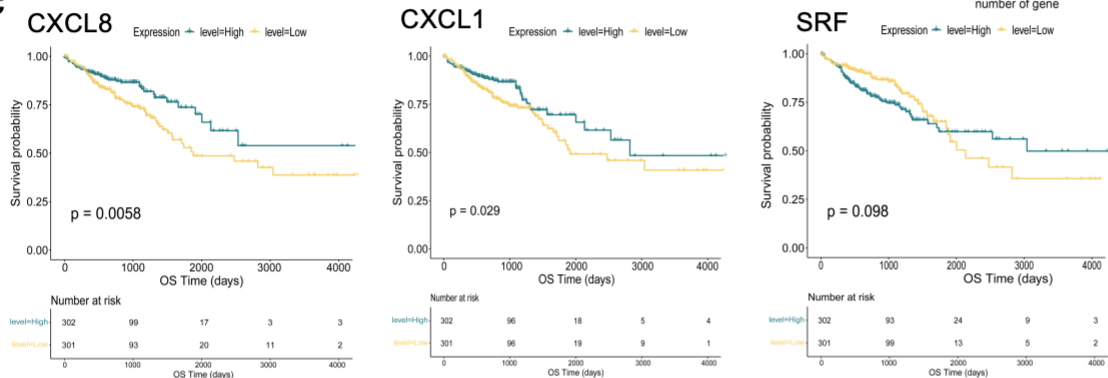**d**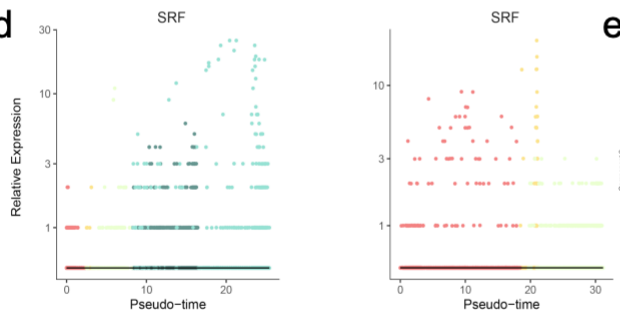**e**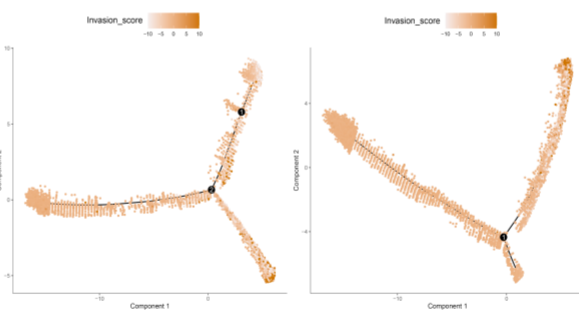**f**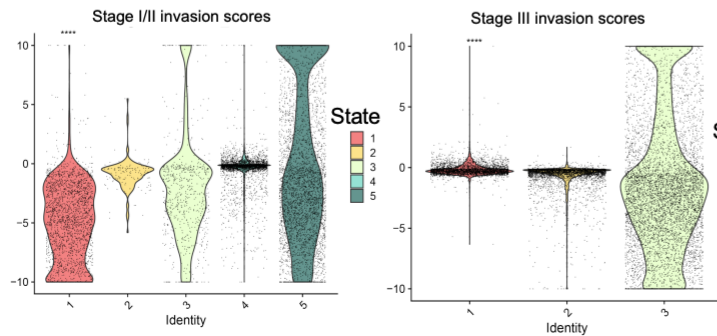**g**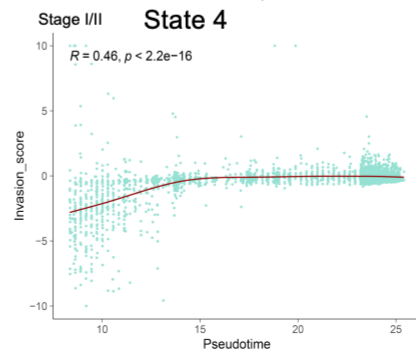

Supplement: Supplementary file 5 — Additional file 5: Cluster cell number statistics. [file 12967_2023_4600_MOESM5_ESM.pdf]

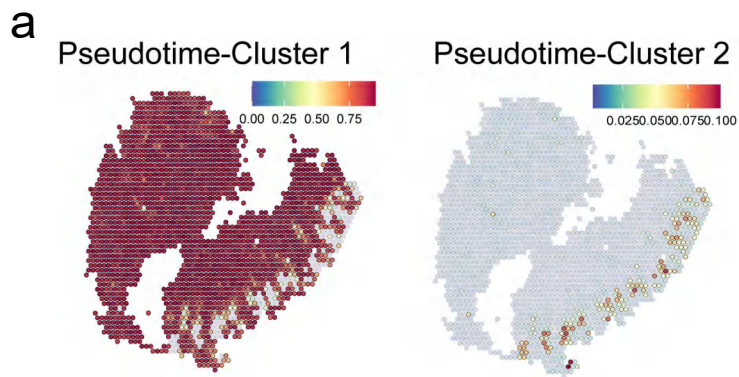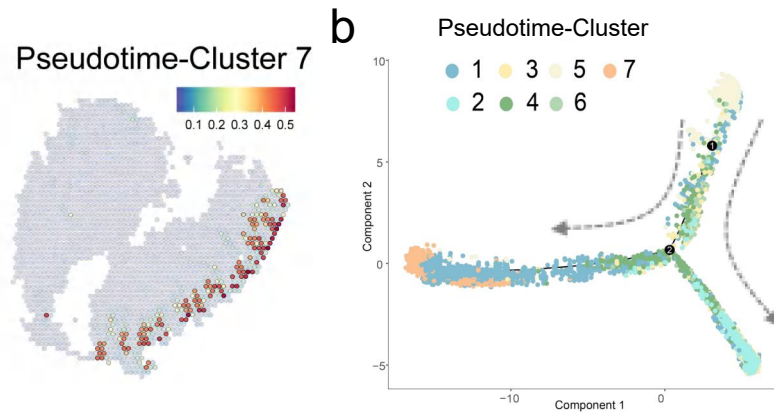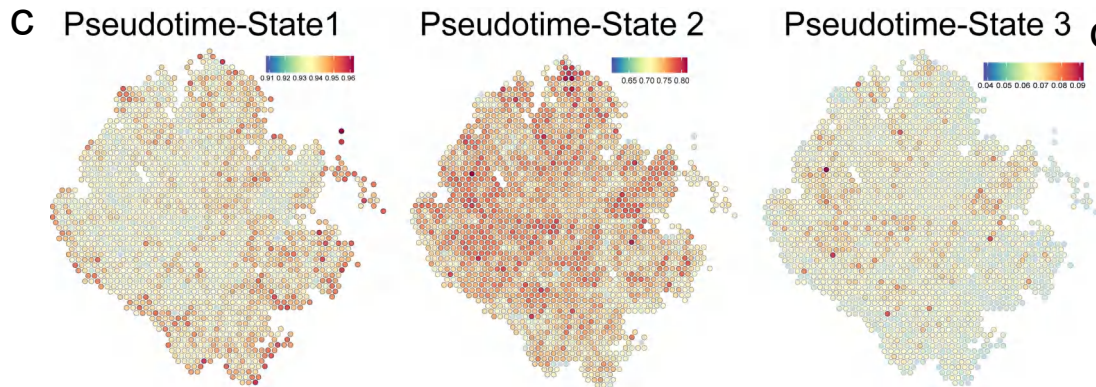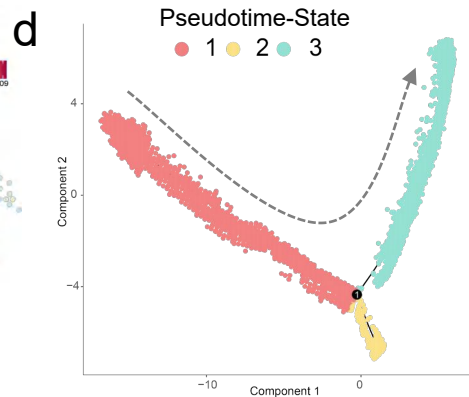

Supplement: Supplementary file 6 — Additional file 6: Marker genes for first annotation. [file 12967_2023_4600_MOESM6_ESM.pdf]

P19\_T

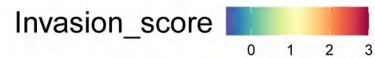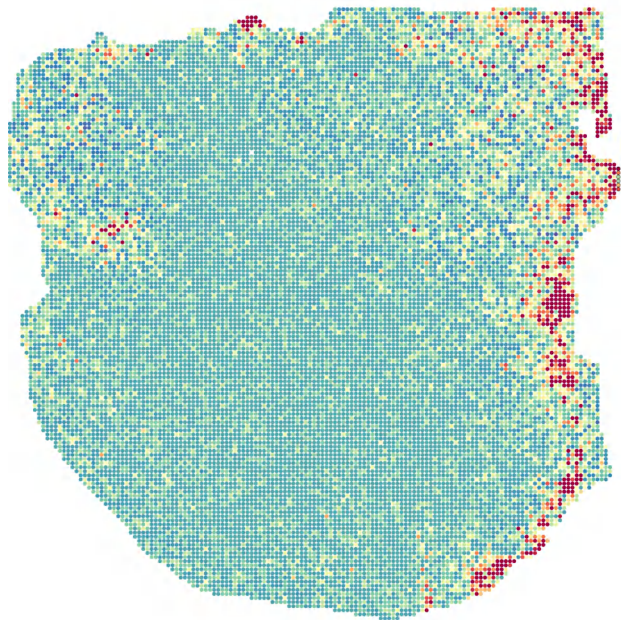

P33\_T

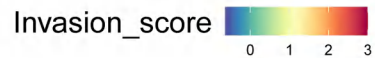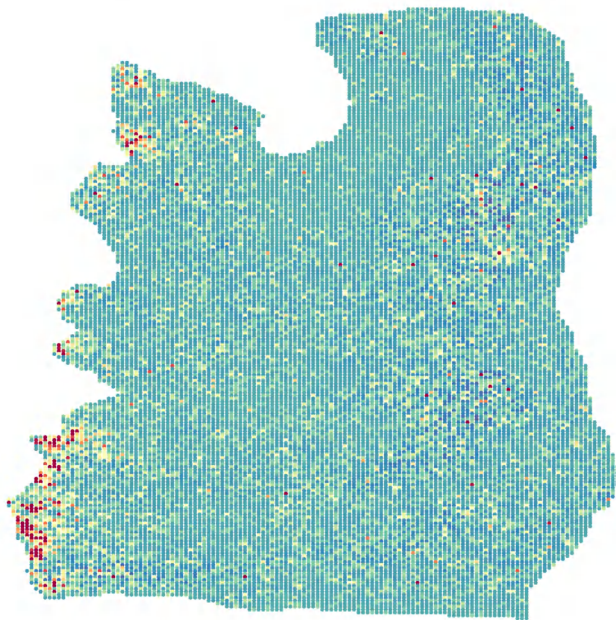

P36\_T

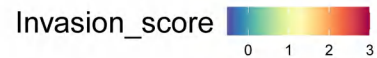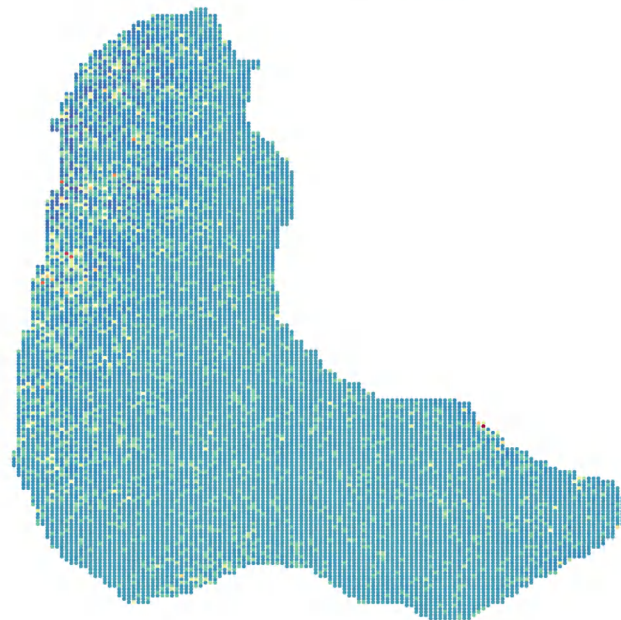

Supplement: Supplementary file 7 — Additional file 7: Marker genes for immune cells. [file 12967_2023_4600_MOESM7_ESM.pdf]

## a Stage I/II

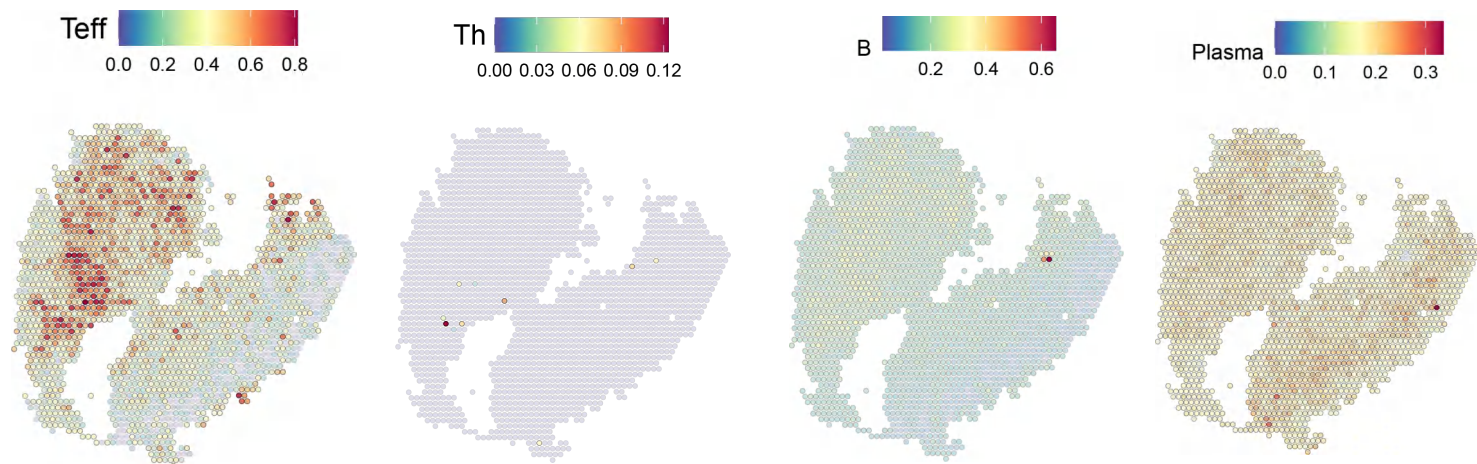

## b Stage III

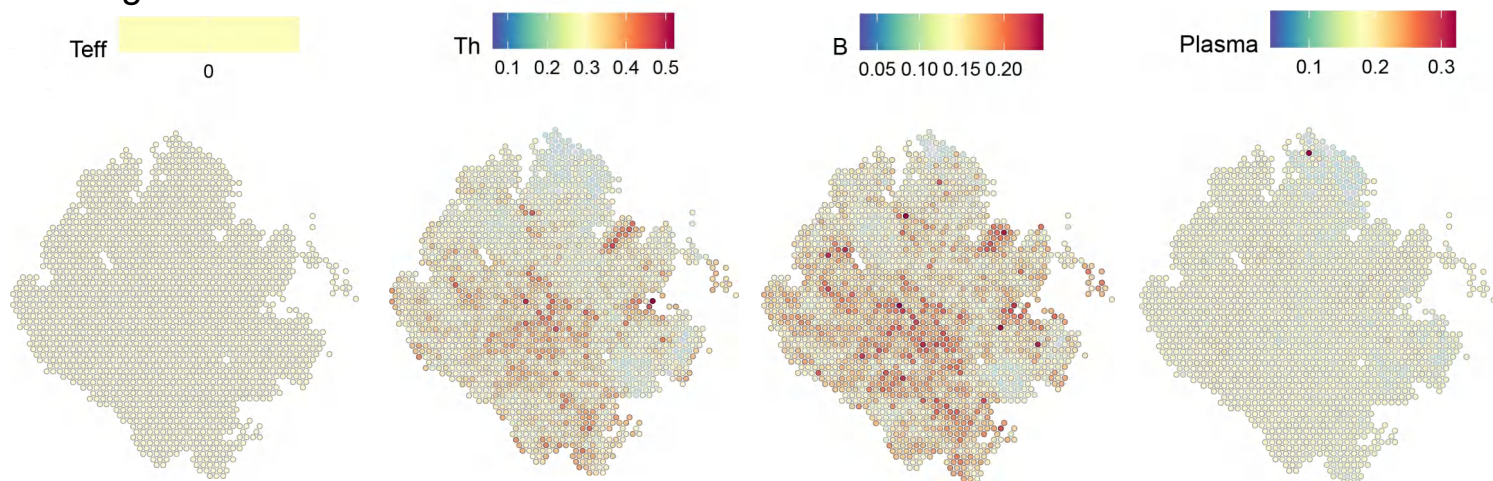

## c

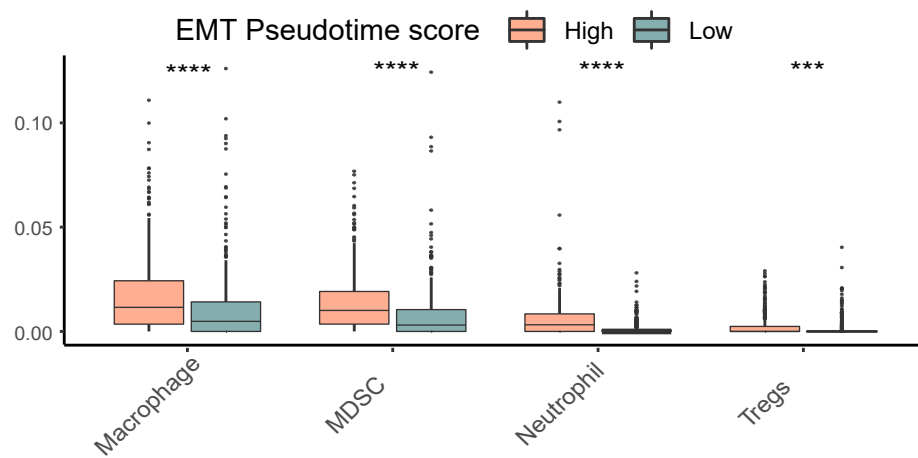

Supplement: Supplementary file 8 — Additional file 8: Marker genes for T cells state. [file 12967_2023_4600_MOESM8_ESM.pdf]

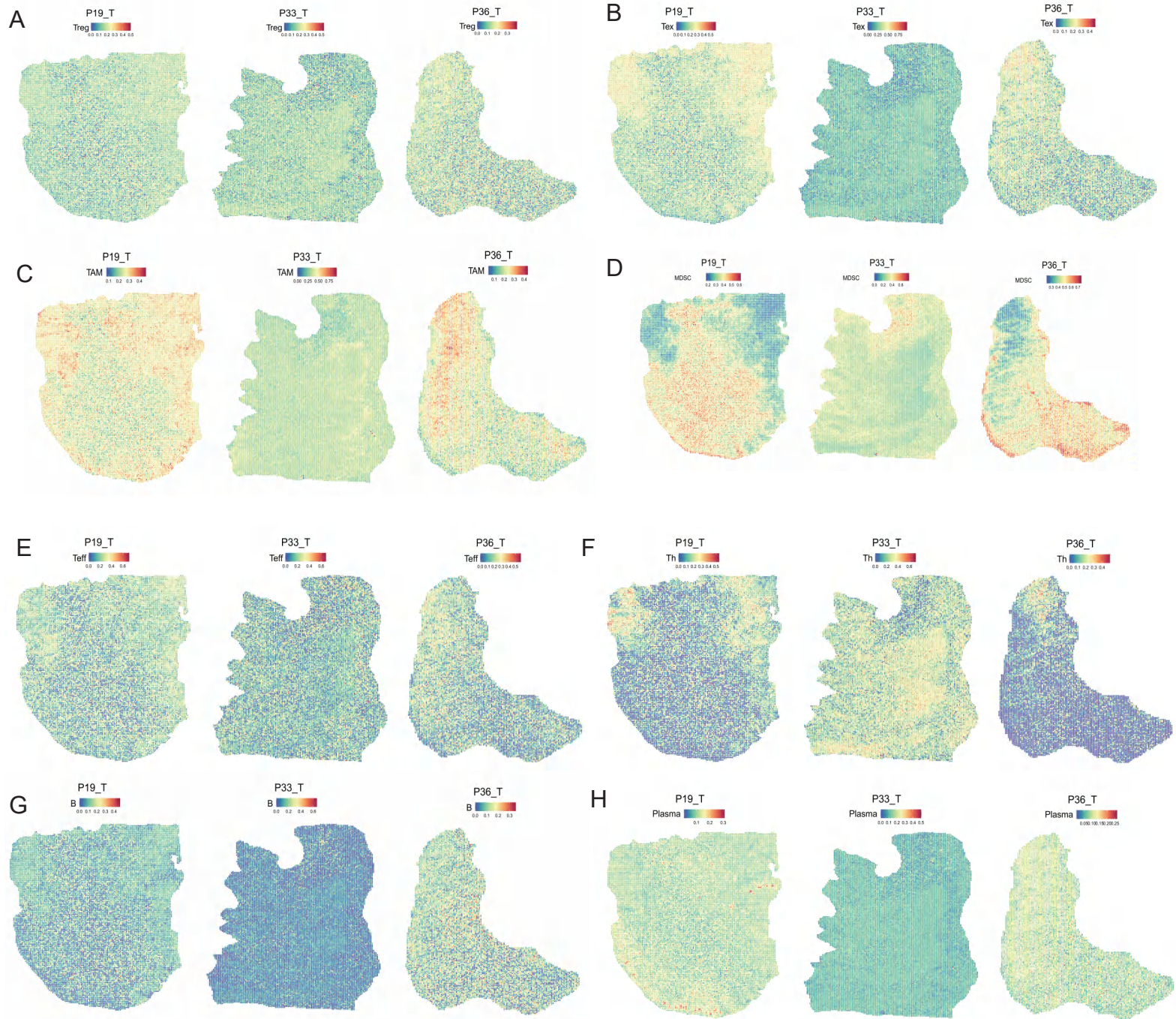

Supplement: Supplementary file 9 — Additional file 9: InferCNV results for Epithelial cells. [file 12967_2023_4600_MOESM9_ESM.pdf]

a

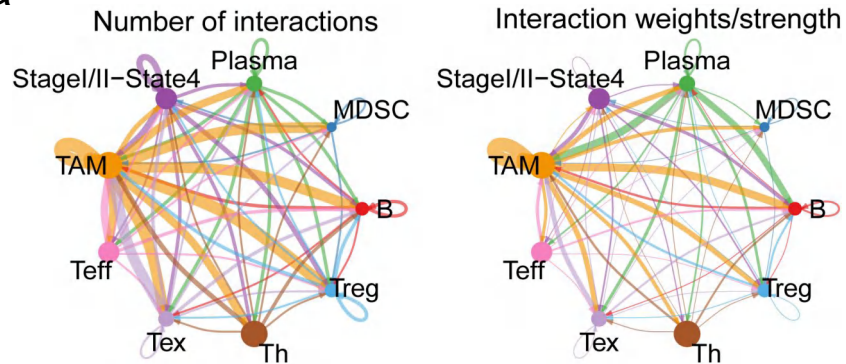

b

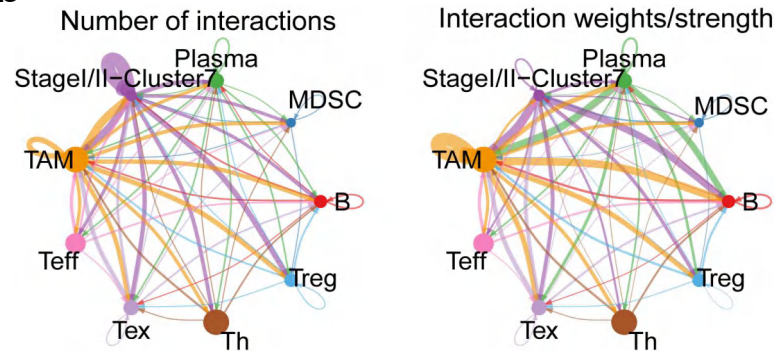

c

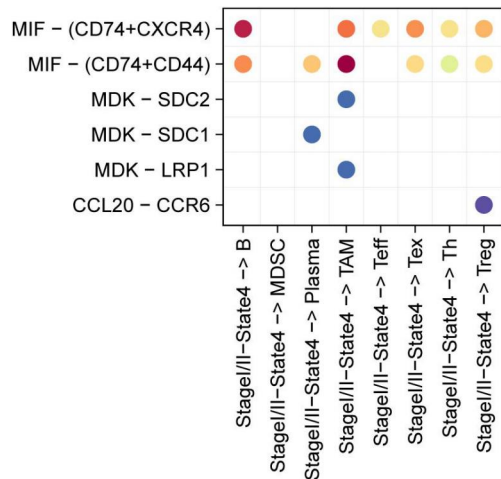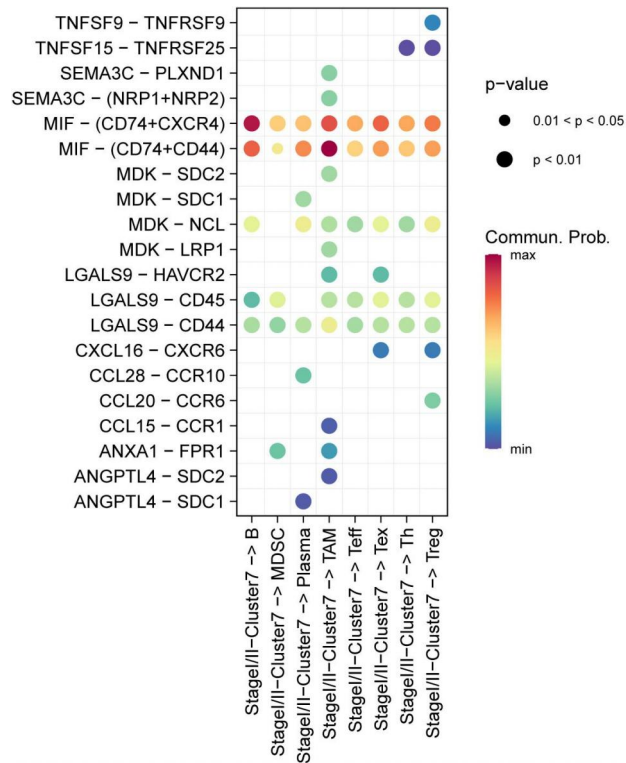

Supplement: Supplementary file 10 — Additional file 10: Top 5 enriched pathways for Epithelial clusters in stage I/II. [file 12967_2023_4600_MOESM10_ESM.pdf]

a

StageII

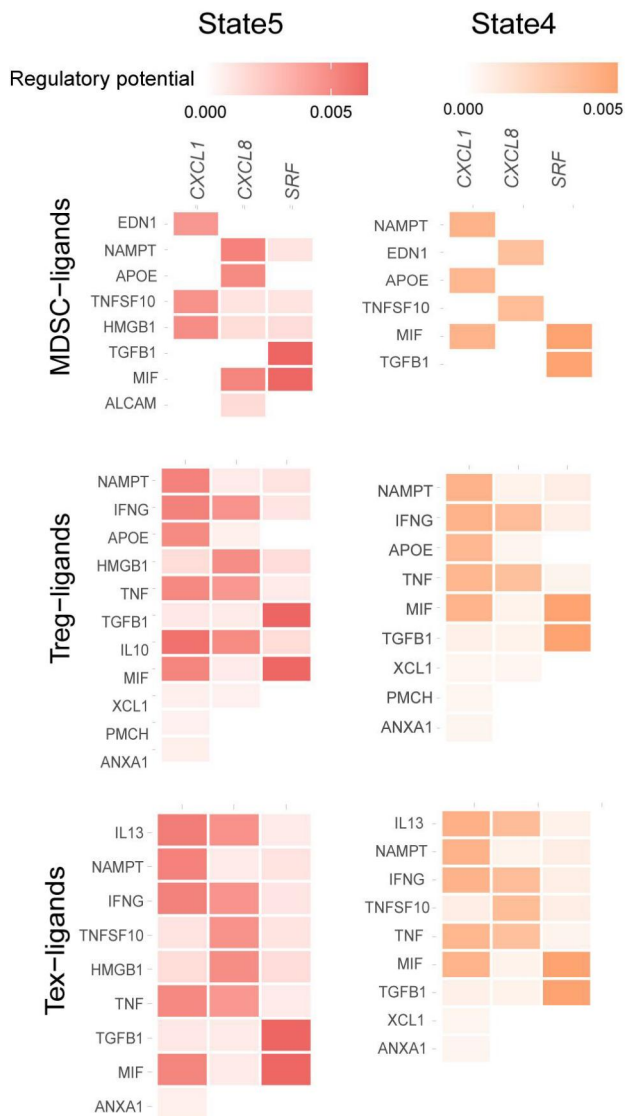

b

StageIII

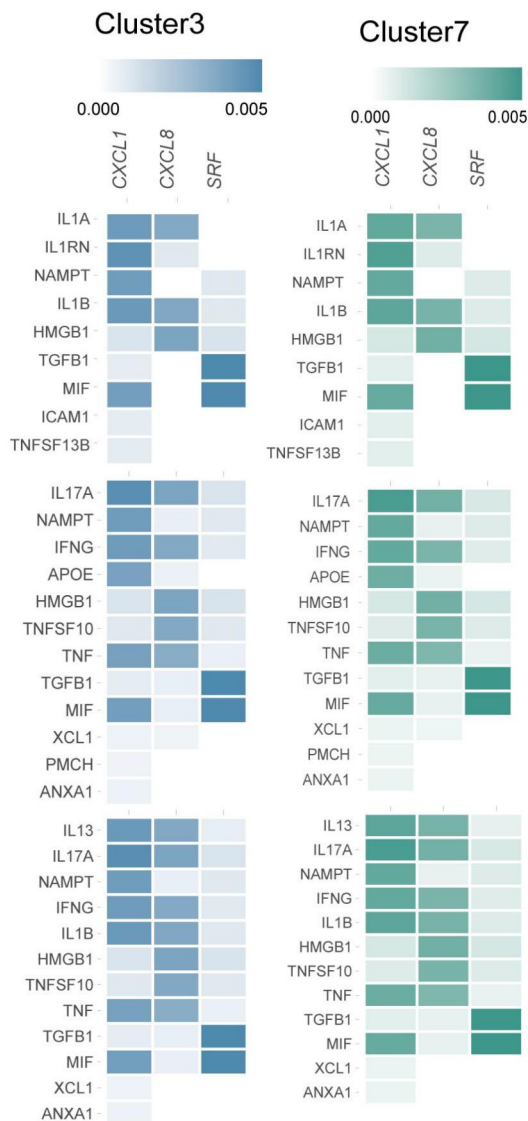

Supplement: Supplementary file 11 — Additional file 11: Top 5 enriched pathways for Epithelial clusters in stage III. [file 12967_2023_4600_MOESM11_ESM.pdf]
